# Principle-based adept predictions of global warming from climate mean states

**DOI:** 10.1093/nsr/nwae442

**Published:** 2024-11-30

**Authors:** Ming Cai, Xiaoming Hu, Jie Sun, Yongyun Hu, Guosheng Liu, Zhaohua Wu, Feng Ding, Wanying Kang

**Affiliations:** Department of Earth, Ocean, and Atmosphere Science, Florida State University, Tallahassee, FL 32306, USA; School of Atmospheric Sciences, Sun Yat-sen University and Southern Marine Science and Engineering Guangdong Laboratory (Zhuhai), Zhuhai 519082, China; Guangdong Province Key Laboratory for Climate Change and Natural Disaster Studies, Sun Yat-sen University, Zhuhai 519082, China; Department of Earth, Ocean, and Atmosphere Science, Florida State University, Tallahassee, FL 32306, USA; Department of Atmospheric and Oceanic Sciences, Peking University, Beijing 100871, China; Department of Earth, Ocean, and Atmosphere Science, Florida State University, Tallahassee, FL 32306, USA; Department of Earth, Ocean, and Atmosphere Science, Florida State University, Tallahassee, FL 32306, USA; Department of Atmospheric and Oceanic Sciences, Peking University, Beijing 100871, China; Department of Earth, Atmospheric, and Planetary Sciences, Massachusetts Institute of Technology, Boston, MA 02139, USA

**Keywords:** global warming, energy balance, energy gain kernel, non-temperature feedback, total climate feedback kernel

## Abstract

Distinguishing anthropogenic warming from natural variability and reducing uncertainty in global-warming projections continue to present challenges. Here, we introduce a novel principle-based framework for predicting global warming from climate mean states that is based solely on carbon-dioxide-increasing scenarios without running climate models and relying on statistical trend analysis. By applying this framework to the climate mean state of 1980–2000, we accurately capture the subsequent global warming (0.403 K predicted versus 0.414 K observed) and polar warming amplification patterns. Our predictions from climate mean states of individual models not only exhibit a high map-correlation skill that is comparable to that of individual Coupled Model Intercomparison Project Phase 6 models for the observed warming, but also capture the temporal pace of their warming under the 1% annual CO_2_-increasing scenario. This work provides the first principle-based confirmation that anthropogenic greenhouse gases are the primary cause of the observed global warming from 1980–2000 to 2000–2020, independently of climate models and statistical analysis.

## Teaser

The novel principles-based framework offers a groundbreaking approach to predicting global warming solely from carbon dioxide increase scenarios without running climate models and performing statistical analysis. By accurately capturing the observed warming and its spatial pattern, this study provides the first principles-based independent confirmation of anthropogenic greenhouse gases as the primary drivers of the warming observed from 1980–2000 to 2000–2020.

## INTRODUCTION

Earth's climate system is undergoing profound changes and the evidence is unequivocal: the atmosphere, oceans and land are all warming, driven by the influence of human activities [1,2]. The impacts of global warming are far-reaching and affect ecosystems, economies and the well-being of communities around the world [3]. Understanding the causes and consequences of this warming is of paramount importance for guiding future climate policy and adaptation efforts [4]. Although climate change is incontrovertible, discerning the underlying drivers of this warming is challenging. Observational records reveal a world that is growing warmer but teasing apart the contributions of natural variability and anthropogenic external forcing remains a complex endeavor [5–11]. Traditional statistical approaches have provided valuable insights, but the intricate interplay of various climatic factors demands more comprehensive methods. Climate models have long been employed to investigate the causes of global warming. However, the inherent complexity of these models, coupled with the wide range of parameterizations that they depend on, introduces uncertainties into their predictions [12–16]. Different models may yield different estimates of future warming [17], leaving room for ambiguity in our understanding of the climate system.

Climate sensitivity studies that use the partial radiative perturbation method [18–22] and the radiative kernel method [23] are thought to hold the promise of leading to principle-based predictions of global warming from climate mean states. However, estimations of climate feedback parameters require data from perturbed climate simulations or climate trend analysis. Moreover, all existing climate feedback analysis frameworks treat the relationships of the temperature feedback with external energy perturbations and with other (i.e. non-temperature) feedback as ‘parallel’ processes, thereby following an addition rule in their mathematical expressions. As a result, none of the existing climate feedback analysis frameworks has yet achieved the capability to predict global warming without running climate models, as they at least require the prior information on temperature changes.

The recent study by Cai *et al.* [24] disentangles the negative and positive aspects of the temperature feedback. The negative aspect corresponds to thermal energy emission perturbations of individual layers, while the positive aspect, represented by the energy gain kernel (EGK), corresponds to the amplification of energy perturbations through radiative thermal coupling within an atmosphere–surface column. Mathematically, the relationships of the positive aspect of the temperature feedback with external energy perturbations and with other feedback follow a multiplication rule rather than an addition rule. The positive aspect of the temperature feedback, encapsulated by the EGK, acts to amplify energy perturbations at an equal rate (for a given location in observations or in a climate model), regardless of their origins, polarity and amplitude. The disentangling of the negative and positive aspects of the temperature feedback rectifies the common misconception in existing climate feedback studies that portray temperature feedback as predominantly negative. In this study, we devise a principle-based framework that allows the adept prediction of global mean warming and its spatial pattern in response to anthropogenic greenhouse gases from climate mean states without running climate models or statistical analysis. Incorporation of the positive aspect of the temperature feedback as a multiplication rule is pivotal for predicting global warming without running climate models.

## RESULTS

### The total climate feedback kernel

The novel principle-based framework is built on our recent discovery of the EGK associated with temperature feedback. Following [25] and [26], the perturbation energy equation within an atmosphere–surface column at a given horizontal location is:


(1) \begin{equation*}
\mathop \sum \limits_{j = 1}^L \left( {\frac{{\partial {{R}_i}}}{{\partial {{T}_j}}}} \right)\Delta {{T}_j} = {\mathrm{\Delta }}F_i^{\left( {\rm EXT} \right)} + \mathop \sum \limits_X {\mathrm{\Delta }}F_i^{\left( X \right)},
\end{equation*}

where $( {{{\bf \Delta }}{\boldsymbol{F}}_{\boldsymbol{i}}^{( {{\boldsymbol{\rm EXT}}} )}} )$ is the vertical profile of external energy perturbations, with *i* = 1 corresponding to the top atmospheric layer and *i* = *L* the surface layer, whereas $( {{{\bf \Delta }}{\boldsymbol{F}}_{\boldsymbol{i}}^{( {\boldsymbol{X}} )}} )$ represents the vertical profile of energy perturbations due to changes in a non-temperature climate variable *X* in response to $( {{{\bf \Delta }}{\boldsymbol{F}}_{\boldsymbol{i}}^{( {{\boldsymbol{\rm EXT}}} )}} )$; $\mathop \sum \nolimits_{\boldsymbol{X}}$ denotes the summation over all non-temperature-feedback values, such as water vapor and cloud feedback; $( {{\boldsymbol{\Delta }}{{{\boldsymbol{T}}}_{\boldsymbol{j}}}} )$ represents the vertical profile of equilibrium temperature changes in response to external energy perturbations; and the matrix $( {\frac{{\partial {{{\boldsymbol{R}}}_{\boldsymbol{i}}}}}{{\partial {{{\boldsymbol{T}}}_{\boldsymbol{j}}}}}} )$ is known as the Planck feedback matrix whose *j*-th column corresponds to the net long-wave (LW) radiative cooling rate (denoted by ‘*R*’) due to 1 K of warming at the *j*-th layer in units of ${\mathrm{W\ }}{{{\mathrm{m}}}^{ - 2}}{{{\mathrm{K}}}^{ - 1}}$ with both *i* and *j* running from 1 for the top layer of an atmosphere–surface column to *L* for the surface layer. As shown in [24], Equation (1) can be rewritten as:


(2) \begin{equation*}
\frac{{\partial {{R}_i}}}{{\partial {{T}_i}}}{\mathrm{\Delta }}{{T}_i} = \mathop \sum \limits_{j = 1}^L {{G}_{i,j}}( {1 + {{\lambda }_{j,j}}} )\Delta F_j^{( {\rm EXT} )},
\end{equation*}

where $( {\frac{{\partial {{R}_i}}}{{\partial {{T}_i}}}} )$ is the diagonal matrix of $( {\frac{{\partial {{R}_i}}}{{\partial {{T}_j}}}} )$, representing the increase in the thermal energy emission of individual layers due to their warming of 1 K; $( {{{G}_{i,\!j}}} ) = ( {\frac{{\partial {{R}_i}}}{{\partial {{T}_i}}}} ){{( {\frac{{\partial {{R}_i}}}{{\partial {{T}_j}}}} )}^{ - 1}}$ is the EGK associated with temperature feedback; and $( {1 + {{\lambda }_{j,\!j}}} )$ with ${{\lambda }_{j,\!j}} = \mathop \sum \nolimits_X \frac{{\Delta F_j^{( X )}}}{{\Delta F_j^{( {\rm EXT} )}}}$ is a diagonal matrix, whose diagonal elements correspond to the multiplication factors of the external energy perturbations by non-temperature feedback at individual layers.

**Figure 1.**
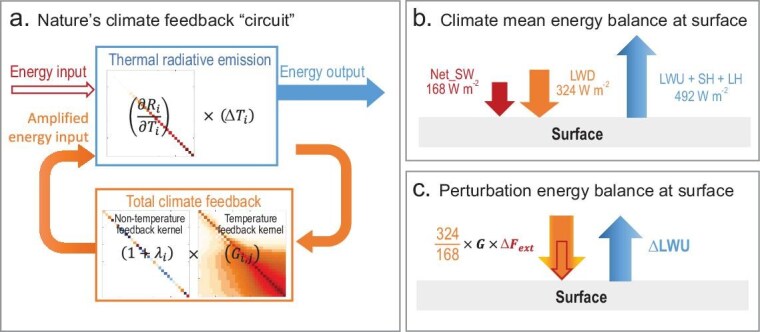
Illustration of nature's climate feedback ‘circuit’. (a) Illustration of nature's climate feedback ‘circuit’, (b) global mean surface energy balance in the climate mean state and (c) perturbation surface energy balance in response to external energy perturbation $\Delta {{F}_{\rm ext}}$. In panel (a), the straight arrow at the top-left, the curved arrows at the bottom, and the straight arrow at the top-right represent the external energy input, amplified external energy input and energy output from Earth's climate system, respectively. The top box represents the thermal radiative emissions of individual layers in an atmosphere–surface column and the bottom box corresponds to the total climate feedback kernel, which equals the product of the energy gain kernel of the temperature-feedback (matrix on the right) and the non-temperature-feedback kernel (matrix on the left). In panel (b), the arrows from the left to the right represent the climate mean total solar energy absorbed by the surface, the downward thermal energy emitted from the atmosphere and the total energy output from the surface equal to the sum of surface thermal energy emission and surface sensible and latent heat fluxes, respectively. The numbers next to the arrows are their climate mean and global mean values taken from Kiehl and Trenberth [31]. In panel (c), the compound downward arrow on the left and the upward arrow on the right represent the total input energy and thermal emission perturbations at the surface, respectively. The strength of the total input energy perturbations equals the product of the input energy perturbation ($\Delta {{F}_{\rm ext}}$), temperature-feedback kernel (*G*) and surface element of the non-temperature-feedback kernel. Both $\Delta {{F}_{\rm ext}}$ and *G* can be calculated from the radiative transfer model by using climate mean state information. The surface element of the non-temperature-feedback kernel is estimated from the amplification of the climate mean solar energy input at the surface by the climate mean greenhouse effect of the atmosphere given in panel (b), whose observed global mean value is 324/168. In panel (b), LWU, SH, and LH represent upward longwave radiative, sensible heat, and latent heat fluxes at surface, respectively.

The matrices inside the blue box and the matrices on the right and left inside the orange box in Fig. 1a, respectively, are representative examples of the matrices $( {\frac{{\partial {{R}_i}}}{{\partial {{T}_i}}}} )$, $( {{{G}_{i,\!j}}} )$ and $( {1 + {{\lambda }_{j,\!j}}} )$. The first two are obtained from a radiative transfer model [27,28] by using only the global mean data of 1980–2000 derived from the ERA5 reanalysis [29] while the third one also uses the data of 2000–2020. It is seen that elements of the diagonal matrix $( {\frac{{\partial {{R}_i}}}{{\partial {{T}_i}}}} )$ are all positive, representing the increase in the thermal energy emission of individual layers in an atmosphere–surface column due to their warming of 1 K. The diagonal elements of the EGK are always greater than unity, corresponding to the amplification through the coupled atmosphere–surface temperature response to the input energy perturbations imposed onto individual layers. The off-diagonal elements of the EGK are all positive, representing the energy gained through the coupled atmosphere–surface temperature response to the input energy perturbations imposed onto individual layers. The strength of the thermal radiation absorption effect that is encapsulated in the EGK is determined collectively by using the climate mean temperature and variables that affect the climate infrared optical thickness, such as water vapor, clouds and surface pressure. Readers are referred to [24] for the physics-based derivations of the EGK and more elaborate discussions on its physical meanings.

According to Equation (2), the external input energy perturbations are subject to two types of feedback amplification and their combined effect obeys a multiplication rule rather than addition/subtraction. The first type is through non-temperature-feedback processes that either enhance the external energy input perturbations at individual layers (i.e. ${{\lambda }_{j,\!j}} > 0$ or positive feedback) or reduce the external energy input perturbations at individual layers (i.e. ${{\lambda }_{j,\!j}} < 0$ or negative feedback). The second type is through the EGK of the temperature feedback. The EGK amplifies the original energy input perturbations at individual layers after their amplification/reduction by non-temperature feedback. Energy perturbations at individual layers are also transferred to neighboring layers through thermal radiative coupling across different layers, leading to energy gains at other layers. We refer to the product of the EGK of the temperature feedback and non-temperature kernels, i.e. $( {{{G}_{i,\!j}}} )( {1 + {{\lambda }_{j,\!j}}} )$, as the total climate feedback kernel.

As illustrated in Fig. 1a, the total climate feedback kernel that is encapsulated in Equation (2) functions in exactly the same way as the first electronic feedback circuit that was invented by H.S. Black in 1934 [30]. The right-hand side (RHS) of Equation (2), or the arrows on the left of the blue box in Fig. 1a, represents the total input energy perturbations into the climate system after accounting for its amplification by the total climate feedback kernel, while the left-hand side (LHS), or the blue arrow, represents the thermal radiative emission perturbations of individual layers that are in radiative equilibrium balance with the total energy perturbations. Although the combined effects of the two types of amplification are imprinted by the role of multiplication, the EGK serves as the most intrinsic part of the total climate feedback kernel. In the absence of non-temperature feedback, the total climate feedback kernel is simply reduced to the EGK of the temperature feedback. The EGK acts to amplify the external input energy perturbations at individual layers after their amplification/reduction by non-temperature feedback and transfers them into other layers via thermal radiative coupling between the different layers in an atmosphere–surface column. In this sense, the total climate feedback kernel is nature's feedback ‘circuit’ within Earth's climate system.

It is important to note that there are subtle yet important differences in the physical meanings of the terms in Equation (1) compared with those in Equation (2), even though both sets of terms represent energy perturbations in units of ${\mathrm{W\ }}{{{\mathrm{m}}}^{ - 2}}$. The LHS of Equation (1) corresponds to the vertical profile of the net LW cooling rate perturbations due to given changes in temperatures. The vertical summation on the LHS of Equation (1) takes into consideration of the vertical thermal radiative coupling of temperature changes in individual layers for the net LW cooling rate perturbations. When using climate mean temperature changes that are derived from climate model simulations or observations, the LHS represents the vertical profile of the net LW cooling rate perturbations at the instant of climate equilblirum, which is in balance with the sum of the energy perturbations due to external forcing and non-temperature feedback at individual layers indicated by the RHS of Equation (1). The equilibrium temperature response to each of the energy perturbation terms on the RHS of Equation (1), or their sum, can be obtained by multiplying the inverse of the Planck feedback matrix by the energy perturbation terms. In a linear sense, this equilibrium temperature response, which is derived via the inverse of the Planck feedback matrix, is equivalent to the solution that is obtained from climate models that adjust temperature solely in response to energy perturbation terms after a long-term integration (e.g. t $\to \infty$). In terms of the climate feedback concept, the net LW cooling rate perturbations due to temperature changes correspond to temperature feedback. Clearly, solving Equation (1) does not reveal how the equilibrium temperature response is achieved. Although the final strength of the temperature feedback can be obtained from the product of the Planck feedback matrix and the equilibrium temperature response, the details of the underlying processes associated with the temperature feedback remain hidden. In other words, because the prior information about temperature changes is necessary to determine the strength of the temperature feedback, the underlying physical processes that are associated with temperature feedback cannot be revealed by using Equation (1). Due to the lack of exciplit or implicit consideration of how the net LW cooling rate perturbations of individual layers at the instant of climate equilblirum are achieved, Equation (1) by itself does not have predictive capability, although it holds the information on how temperature changes contribute to the energy balance for a perturbed climate state, as indicated by the summation of the individual energy perturbations.

Compared with Equation (1), the vertical summation appears on the RHS of Equation (2). This summation, when it appears on the RHS of the perturbation energy equations, takes into consideration the amplification of given energy perturbations (external or due to non-temperature feedback) by temperature feedback via vertical thermal radiative coupling. Because the EGK is obtained via the inverse of the Planck feedback matrix, it represents the analytical (linearized) equilibrium solution of climate models in response to input energy perturbations, considering only the temperature feedback. The equilibrium solution that is represented by the EGK is expressed as the amplification of the input energy perturbations at the layers where the input energy is located and gains of energy by other layers rather than the equilibrium temperature response. The amplified energy perturbations correspond to the end effect of the temperature feedback. It is the vertical thermal radiative coupling due to temperature feedback that makes the information of the original energy perturbations at individual layers propagate to other layers. The energy propagation between individual layers is mathematically represented by the vertical summation, accounting for both the amplification of the original energy perturbations at individual layers and the energy that is gained at other layers. Therefore, the EGK contains information that elucidates the underlying physical processes that are associated with temperature feedback. It directly determines the amplification rate of the temperature feedback in response to any energy perturbations under consideration (external or due to non-temperature feedback) without requiring prior knowledge of the input energy perturbations (i.e. the strength, vertical distribution and sign) nor their equilibrium temperature responses. In this sense, the EGK retains predictive capability for the amplification rate of any given input energy perturbations by temperature feedback.

The RHS of Equation (2) also indicates that the relationship of the temperature feedback with the external energy perturbations and with other feedback follows a multiplication rule rather than an addition rule and its amplification of the energy perturbations occurs at an equal rate (for a given location in observations or in a climate model), regardless of their origins, polarity and amplitude. As a result, the perturbation energy equation written in the form of Equation (2) involves only two terms. One term, given on the LHS, corresponds to the emission perturbations from individual layers, acting as sinks for energy perturbations. The other term, given on the RHS, represents the amplified energy perturbations due to the temperature feedback, with energy perturbations from non-temperature sources being additive. Because the EGK is dervied solely from the climate mean state in the absence of external energy perturbations, one can directly predict the final amplifications of any given energy perturbations by the temperature feedback alone from the RHS of Equation (2) without needing to know the actual temperature changes that are derived from climate model simulations or observations. The temperature changes at individual layers can then be determined by ensuring that the emission perturbations from each layer balance with the amplified energy perturbations at that layer, corresponding to the LHS of Equation (2). Therefore, the rewriting of Equation (1) as Equation (2) transforms the perturbation energy equation from a diagnostic equation to a prognostic equation when energy perturbations are given.

### Principle-based prediction framework for global warming

The EGK can be determined from the climate mean states by using a standard radiative transfer model. For a given CO_2_-increasing scenario, the external energy input can also be calculated from climate mean states by using the same radiative transfer model (see Supplementary Text 1 for the methods). However, one cannot directly apply the total climate feedback kernel that is illustrated in Fig. 1a to anthropogenic radiative forcing for predicting global warming because the non-temperature-feedback kernel, namely $(1 + {{{\boldsymbol{\lambda }}}_{{\boldsymbol{j}},{\boldsymbol{\! j}}}})$, cannot be determined without running climate models or using observed trends.

We have devised a novel approach to transform Equation (2) from a partially predictive function into a fully predictive function for global surface warming in response to anthropogenic radiative forcing from climate mean states without prior information regarding $(1 + {{\lambda }_{j,\!j}})$ from climate simulations or observations. This transition is achieved by estimating the strength of the non-temperature feedback at the surface from the climate mean surface energy balance equation, as illustrated in Fig. 1b and c. Specifically, the CO_2_-induced surface temperature change $\Delta {{T}_s}$ can be determined from:


(3) \begin{equation*}
\Delta {{T}_s} = \frac{{{{A}_{\rm NT}}\mathop \sum \nolimits_j {{G}_{s,j}}\Delta F_j^{\left( {\rm EXT} \right)}}}{{4\sigma {\overline{T}}_s^3}},
\end{equation*}

where σ is the Stefan–Boltzmann constant, $\overline {{{T}_s}}$ is the climate mean surface temperature, *G*_s,j_ corresponds to the surface elements of the EGK and $\mathop \sum \nolimits_j {{G}_{s,j}}\Delta F_j^{( {\rm EXT} )}$ is the surface component of the amplified external energy perturbation by the EGK of the temperature feedback. In Equation (3), *A*_NT_ represents the surface element of the non-temperature-feedback kernel (i.e. the term 1 + *λ*_s_) of the diagonal matrix in the bottom left corner of Fig. 1a. As indicated in Equations (8)–(12) in Supplementary Text 2, *A*_NT_ can be estimated from the climate mean state according to:


(4) \begin{equation*}
{{A}_{\rm NT}} \approx \frac{{\overline{R}_s^ \downarrow }}{{\overline{S}_s^ \downarrow - \overline{S}_s^ \uparrow }},
\end{equation*}

where $\overline R _s^ \downarrow$, $\overline S _s^ \downarrow$ and $\overline S _s^ \uparrow$ are the climate mean downward LW radiative fluxes at the surface and the downward and upward solar energy fluxes at the surface, respectively.

In essence, the key step of this new approach is to extract information on the non-temperature-feedback kernel at the surface (i.e. *A*_NT_) from the climate mean surface energy balance equation by adopting reanalysis or model simulations. In the climate mean state, the global mean of *A*_NT_ defined in Equation (4) measures the amplification of the net solar energy input to the surface by the atmospheric greenhouse effect, as illustrated in Fig. 1b. Such amplification varies spatially due to spatial variations in water vapor, clouds, ice/snow coverage and energy transport convergence/divergence by atmospheric circulations. Building upon this understanding, we can consider the external energy input to the surface that is amplified by the temperature feedback (*G* × ∆*F*_ext_ in Fig. 1c) for the perturbed climate state to be equivalent to the net solar energy input (Net_SW in Fig. 1b) to the surface in the climate mean state. Similarly, *A*_NT_ acts as non-temperature feedback to the energy input perturbation at the surface for a perturbed climate state, just as it amplifies the total solar energy input at the surface in the climate mean state. In light of the above, the product of *A*_NT_ that is derived from the climate mean state and the external energy input amplified by the temperature feedback (*A*_NT_ × *G* × ∆*F*_ext_ in Fig. 1c) is the total energy perturbation at the surface that is initiated by the external energy input. The energy balance between the total energy perturbations at the surface and the enhanced thermal emission from the surface (∆LWU in Fig. 1c) determines the global surface warming in response to increasing CO_2_, including its spatial pattern, as expressed in Equation (3).

In Supplementary Text 2, it is demonstrated that the *A*_NT_ that is obtained by using Equation (4) from climate mean states is highly correlated (∼0.9, Fig. S1) with the counterparts that are directly diagnosed from perturbed climate simulations by using Equation (11). Note that the *A*_NT_ that is obtained by using Equation (11) involves actual energy perturbations due to the non-temperature feedback for calculating ${{\lambda }_{j,\!j}} = \mathop \sum \nolimits_X \frac{{\Delta F_j^{( X )}}}{{\Delta F_j^{( {\rm EXT} )}}}$, where $\Delta F_j^{( X )}$ includes both radiative energy perturbations due to changes in water vapor, clouds and surface albedo, and non-radiative energy perturbations due to changes in atmospheric convective and advective processes that are derived from perturbed climate simulations. Therefore, the high correlation between the *A*_NT_ that is obtained from climate mean states and the *A*_NT_ that is diagnosed from actual energy perturbations due to non-temperature feedback directly validates Equation (4) for estimating *A*_NT_ without running climate models.

The extraction of the *A*_NT_ from climate mean states rather than from running climate models and a reliance on the statistical trend analysis of non-temperature-feedback variables, such as clouds, water vapor and ice/snow coverage, enables the transition of the feedback circuit, as illustrated in Fig. 1a, from a diagnostic tool into a prediction tool for global warming. The global mean value of *A*_NT_ is well recognized in introductory climate textbooks for illustrating the greenhouse effect of Earth's climate system and its spatial pattern can be easily determined from climate mean states. Despite its familiarity and easy access, the true significance and role of *A*_NT_ in retaining crucial information about surface energy amplification by non-temperature feedback for predicting the response of global warming to external energy perturbations remained unknown until our findings. In essence, we utilize the *A*_NT_ that is derived from the climate mean state to infer the multiplication factor of external energy perturbations at the surface by using non-temperature feedback for a perturbed climate state.

### Prediction of observed global warming from 1980–2000 to 2000–2020

**Figure 2.**
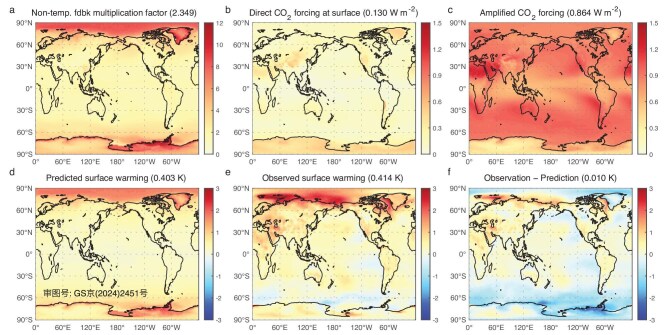
Prediction of observed global warming from the 1980–2000 mean state. Maps of (a) multiplication factor by non-temperature feedback (*A*_NT_) derived from the 1980–2000 time mean state (dimensionless), (b) external energy perturbations at the surface (W m^−2^) determined from the observed increase in the CO_2_ concentration from 1980–2000 to 2000–2020, (c) amplified external energy perturbations at the surface (W m^−2^) through the energy gain kernel of the temperature feedback, (d) predicted global warming (K), (e) the observed warming (K) and (f) the difference between panels (e) and (d). The numbers inside ‘()’ in the title are their global mean values.

We calculate the *A*_NT_ according to Equation (4) by using the 1980–2000 mean data that were derived from the ERA5 reanalysis [29]. Figure 2a reveals significant spatial variation in the observation-derived *A*_NT_, which varies from generally low values in the tropics, with the lowest possible value close to 1 in the eastern Pacific cold tongue region, to notably much higher values of >9 in polar regions. The smaller values of *A*_NT_ in the tropics reflect the partial cancellation of strong positive water vapor feedback, net negative cloud short-wave (SW) and LW feedback, and strong negative feedback due to surface evaporation, vertical convective processes and energy flux divergence that is associated with atmospheric poleward energy transport [32–34]. The much larger values of *A*_NT_ at high latitudes reflect both the dominance of positive feedback processes, such as water vapor, ice-albedo and LW cloud feedback, plus the energy flux convergence that is associated with poleward atmospheric energy transport and the lack of negative feedback processes, such as SW cloud feedback and vertical convections [32,35,36]. The dominance of positive feedback over high latitudes explains the meridionally decreasing profile of the ratio of downward LW fluxes to the net solar energy fluxes at the surface. Eastern Antarctica, with its higher elevation, exhibits both lower surface pressure and colder temperatures. Their combined effect is a drier atmosphere with fewer clouds, resulting in smaller *A*_NT_ values than those over Western Antarctica.

We use the radiative transfer model to compute the external radiative forcing at the surface based on the observed increase in CO_2_ concentrations from 352.2 ppm in 1980–2000 to 385 ppm in 2000–2020 (Fig. 2b) and its amplification through the EGK of the temperature feedback (Fig. 2c). The spatial pattern of the CO_2_-induced radiative forcing at the surface is largely shaped by the scarcity of climate mean moisture in the atmosphere. For the same increase in the CO_2_ concentration, the percentage change in the atmospheric opacity is greater over regions where atmospheric moisture is scarce, such as highly elevated areas, cold places and deserts, resulting in stronger external radiative forcing at the surface [37–39]. The EGK serves to amplify the external energy perturbations at the surface and transfer the external energy perturbations in the atmosphere to the surface layer through temperature feedback, as depicted in Fig. 1a. Over the regions where atmospheric moisture is scarce, the relatively strong positive external energy perturbations at the surface are suppressed through temperature feedback. In regions where climate mean atmospheric moisture and/or low-level clouds are abundant, the vertical extent of the positive external energy perturbations that are induced by increasing CO_2_ extends from the surface [37,39]. As a result, the temperature-feedback-amplified CO_2_-induced energy perturbations at the surface tend to be more pronounced in regions where climate mean atmospheric moisture and/or low-level clouds are abundant, such as the Southern Ocean; the west coasts of California, Peru and Chile; and the Arctic Ocean. In addition to these regions, the values of the EGK are also greater over places where the climate mean temperature is high, such as the Sahara Desert, resulting in a stronger amplification of CO_2_-induced radiative forcing. Overall, the positive temperature-feedback loop through the thermal radiative coupling of the atmosphere surface plays a critical role in amplifying the CO_2_-induced radiative forcing at the surface, increasing it 6.6-fold from a global mean value of 0.13 to 0.86 W m^−2^.

**Figure 3.**
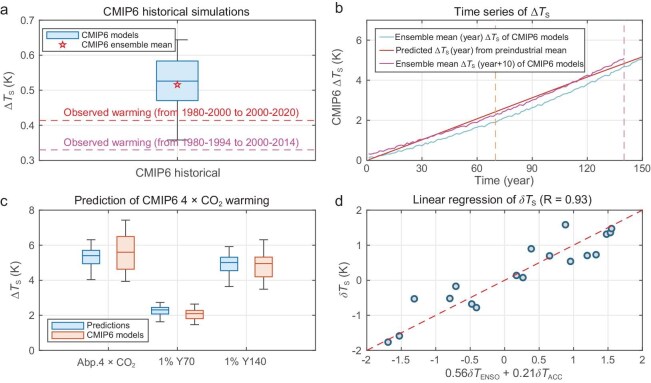
Summary of skill evaluations. (a) Box-and-whisker plot of the observed global mean warming (K, dashed line) predicted by the 18 CMIP6 historical simulations, (b) yearly time series of global mean warming (K) under the 1% annual CO_2_-inceasing scenario predicted from the ensemble-mean pre-industrial climate state and original CMIP6 ensemble-mean warming projection, (c) box-and-whisker plots of global mean warming (K) predicted from pre-industrial (1900–1910) climate states versus those from the 18 CMIP6 models, (d) scatter plot of global mean values of Fig. S9A–R (K, ordinate) versus $0.56\delta {{T}_{{ENSO}}} + 0.21\delta {{T}_{ACC}}$ (K, abscissa), where $\delta {{T}_{{ENSO}}}$ corresponds to the mean values of the data over the black box (170°W–120°W, 5°S–5°N) and $\delta {{T}_{ACC}}$the mean values of the data over the red box (55°S–65°S) in Fig. 4f and in Fig. S9A–R. In (b), the magenta line is otherwise identical to the blue line except that the former is shifted 10 years ahead for a visual illustration of the delay of the continuous transient response to the 1% annual CO_2_ increase. In (a) and (c), the central mark for each box indicates the median, and the bottom and top edges indicate the 25th and 75th percentiles, respectively. The whiskers extend to the most extreme data points. To remove the transient response signal, we adjust the values of the blue box for the 1% annual CO_2_ increase by the same constant value (0.40 K) such that their ensemble-mean warming is the same as the CMIP6 ensemble mean at Year 140.

The product of panels (a) and (c) of Fig. 2, divided by the Stefan–Boltzmann feedback parameter (i.e. $4\sigma \overline{T}_s^3$), yields our predictions for the surface temperature changes from 1980–2000 to 2000–2020 (Fig. 2d). Our predicted global warming is solely attributed to CO_2_-induced radiative forcing and its amplification by the product of the EGK of the temperature feedback and the non-temperature feedback. A comparison of Fig. 2d with Fig. 2e reveals that our prediction nearly perfectly reproduces the observed global mean warming (0.403 K compared with the observed 0.414 K). Our prediction of the observed global mean warming compares favorably to the predictions by 18 CMIP6 (Coupled Model Intercomparison Project Phase 6; see Table S1 for a list of these models) historical simulations [40]. As shown in Fig. 3a, the global mean warming from 1980–1994 to 2000–2014 that was captured by the ensemble-mean CMIP6 historical simulation is 0.516 K, with a median global mean warming of 0.526 K. The observed warming from 1980–1994 to 2000–2014 is 0.332 K. Therefore, our prediction has a much smaller error in terms of the global mean warming. In terms of spatial patterns, the predicted warming exhibits amplified warming over the Arctic and along the coastal areas of Greenland and the Antarctic continent, with generally weaker warming over tropical latitudes, indicating an evident resemblance to their observational counterparts (Fig. 2e).

### Reproduction of climate model global-warming projections

**Figure 4.**
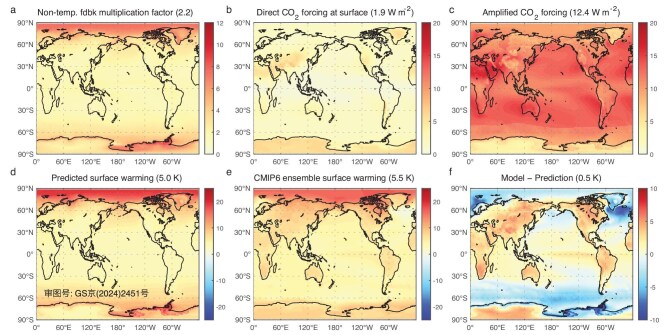
Prediction of global warming from the CMIP6 ensemble-mean pre-industrial climate state. Maps of (a) multiplication factor by non-temperature feedback (*A*_NT_) derived from the pre-industrial mean state of the CMIP6 models ensemble mean (dimensionless), (b) external energy perturbations at the surface (W m^−2^) under the abrupt 4 × CO_2_ scenario, (c) amplified external energy perturbations at the surface (W m^−2^) through the energy gain kernel of the temperature feedback, (d) predicted global warming (K), (e) the CMIP6 models ensemble-mean warming projection under the 4 × CO_2_ scenario (K) and (f) the difference between panels (e) and (d). The numbers inside ‘()’ in the title are their global mean values.

To further assess the prediction ability of the principle-based framework, we made global-warming predictions from pre-industrial climate mean states that were simulated by each of the 18 CMIP6 models. The spatial pattern of the *A*_NT_ that is derived from the ensemble-mean pre-industrial climate state of these 18 models (Fig. 4a) is quite comparable to that of their observational counterparts (Fig. 2a), except that its global mean is ∼10% weaker (2.2 versus 2.4). The spatial patterns of the CO_2_-induced direct radiative forcing (Fig. 4b) and its amplification by temperature feedback (Fig. 4c) are also very close to their observed counterparts, as shown in Fig. 2, except for the much larger (∼14.5 times) values, which is expected from the quadrupling of CO_2_ (4 × CO_2_) instead of the 9% increase in CO_2_. In particular, the global mean amplification rate of CO_2_-induced radiative forcing by temperature feedback (12.4/1.9 = 6.5) is nearly identical to that of its observational counterpart (0.87/0.13 = 6.7). Our predictions from the pre-industrial mean climate state (Fig. 4d) closely capture the original ensemble-mean warming projection of CMIP6 models (Fig. 4e) under the abrupt 4 × CO_2_ scenario, which is ∼0.4 K weaker than the ensemble-mean warming of CMIP6 models (5.1 versus 5.5 K). The new approach can also predict the temporal evolution of climate response to a temporally evolving increase in CO_2_. As illustrated in Fig. 3b, our global-warming prediction (red line) captures the temporal pace of the original ensemble-mean warming projection from the CMIP6 models under the 1% annual CO_2_ increase scenario (blue line). Since our prediction is based on climate equilibrium states, a delay of 10 years (magenta line) or longer of CMIP6 model simulations from our predictions is expected.

The results of the warming projections of individual CMIP6 models and our predictions from their pre-industrial climate mean states are given in Figs S2–S6 for the abrupt 4 × CO_2_ scenario and in Fig. S7 for the 1% annual CO_2_ increase scenario. The box-and-whisker plots (Fig. 3c) show that our global mean warming predictions capture the median value of the CMIP6 projections nearly exactly. The normalized mean absolute differences (NMAD) between our global mean predictions and the CMIP6 projections are <20% of the model ensemble-mean warming (see the definition of NMAD in Equation (7) of Text S1 and the values of NMAD shown in Table S1). This is noticeably smaller than the global mean error of the ensemble mean of the CMIP6 historical simulations for the observed global warming (∼57% = (0.52−0.33)/0.33, Fig. 3a). Furthermore, the inter-model spread of our global mean warming predictions is noticeably smaller and tends to be less dependent on the strength of the external forcing compared with the CMIP6 projections. This suggests that a significant part of the CMIP6 inter-model spread is due to the inter-model spreads of non-temperature feedback rather than the inter-model spread of their climate mean states.

### Oceanic response to CO_2_ forcing

By design, our global-warming prediction captures only the surface temperature response to external energy perturbations, with temperature and non-temperature feedback in atmosphere–surface columns taken into consideration. Therefore, differences between the observed or simulated warming and our predictions may be regarded as oceanic responses to external forcings plus internal climate variability. Figure 2f shows that the difference between the observed trends from 1980–2000 to 2000–2020 and our prediction is dominated by signals from the negative phase of the Pacific Decadal Oscillation (PDO) mode [9,41] and the oceanic response to CO_2_-induced radiative forcing. The latter is evident from the general weak cooling over the Arctic Ocean and North Atlantic Ocean and the weak warming over the South Atlantic Ocean, indicating the weakening trend of the Atlantic Meridional Overturning Circulation (AMOC) in response to CO_2_-induced radiative forcing [42,43]. Additionally, the general cooling over the Southern Ocean surrounding the Antarctic continent shelf is indicative of stronger oceanic upwelling along the Antarctic circumpolar current (ACC) in response to CO_2_ forcing [44].

The differences between the CMIP6 projections and our predictions (Fig. 4f, Figs S8A–R and S9A–R) reveal that all CMIP6 models can capture the observed strengthening response of oceanic upwelling over the Southern Oceans and the weakening of AMOC in response to increasing CO_2_ [45], albeit with varying degrees of strength. The map correlation between Fig. 4f and Fig. 2f over the Southern Ocean is as high as 0.63 and that between each of Fig. S8A–R (or Fig. S9A–R) and Fig. 2f ranges from 0.32 to 0.68 (not shown here), confirming that all CMIP6 models capture the observed cooling response of the Southern Ocean to CO_2_ forcing. In addition to the cooling response of the Southern Ocean, all CMIP6 model simulations also show a strengthening response of El Niño events in their global-warming projections [46]. The strengthening response of El Niño events in individual CMIP6 models is positively correlated (0.41) with the surface temperature response along the ACC (Fig. S10C), indicating that models with a stronger El Niño response would also experience a weaker upwelling response along the ACC [47]. The inter-model spread of the strengthening response of El Niño contributes to the inter-model spread of the CMIP6 global-warming projections (Fig. S10A), as does the inter-model spread of the strengthening response of the oceanic upwelling along the ACC (Fig. S10B). The presence of these two distinct oceanic responses to abrupt changes of 4 × CO_2_ in the CMIP6 models explains most of the differences (86.5%) in the global-warming projections of the CMIP6 models from our predictions (Fig. 3d).

### Skill comparison of our predictions with CMIP6 historical simulations

**Figure 5.**
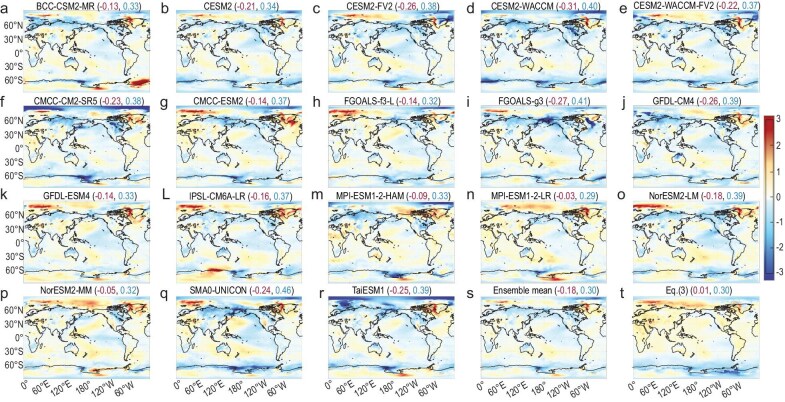
Difference between the observed surface warming and predictions (K). (a–r) Individual CMIP6 historical simulations, (s) the ensemble mean of CMIP6 historical simulations and (t) our predictions. In (a–s), the observed warming is defined as the surface temperature difference between 2000–2014 and 1980–1994 derived from the ERA5 reanalysis, which are consistent with the warming predictions by CMIP6 historical simulations for the same periods. In (t), the observed warming is defined as the surface temperature difference between 2000–2020 and 1980–2000 derived from the ERA5 reanalysis, consistently with the CO_2_ increase from 1980–2000 to 2000–2020 considered in our prediction. The numbers in the parentheses in the title represent the global mean values (K) and global mean absolute errors (K), respectively. Note that the red numbers correspond to the global mean errors, with their signs reversed.

Aside from excluding the oceanic response to CO_2_ forcing in our prediction, our results noticeably underestimate warming over land but overestimate warming of the Arctic Ocean (Figs 2f, 4f and Fig. S9). Figure 5 reveals that, in addition to overestimating the observed global mean warming, the CMIP6 historical simulations also tend to overestimate the warming over the Arctic Ocean. However, they do not show a systematic underestimation of warming over land. The global mean absolute error of our prediction for the observed warming is ∼0.30 K, which is comparable to the values from the CMIP6 historical simulations, which range from 0.30 to 0.46 K.

**Figure 6.**
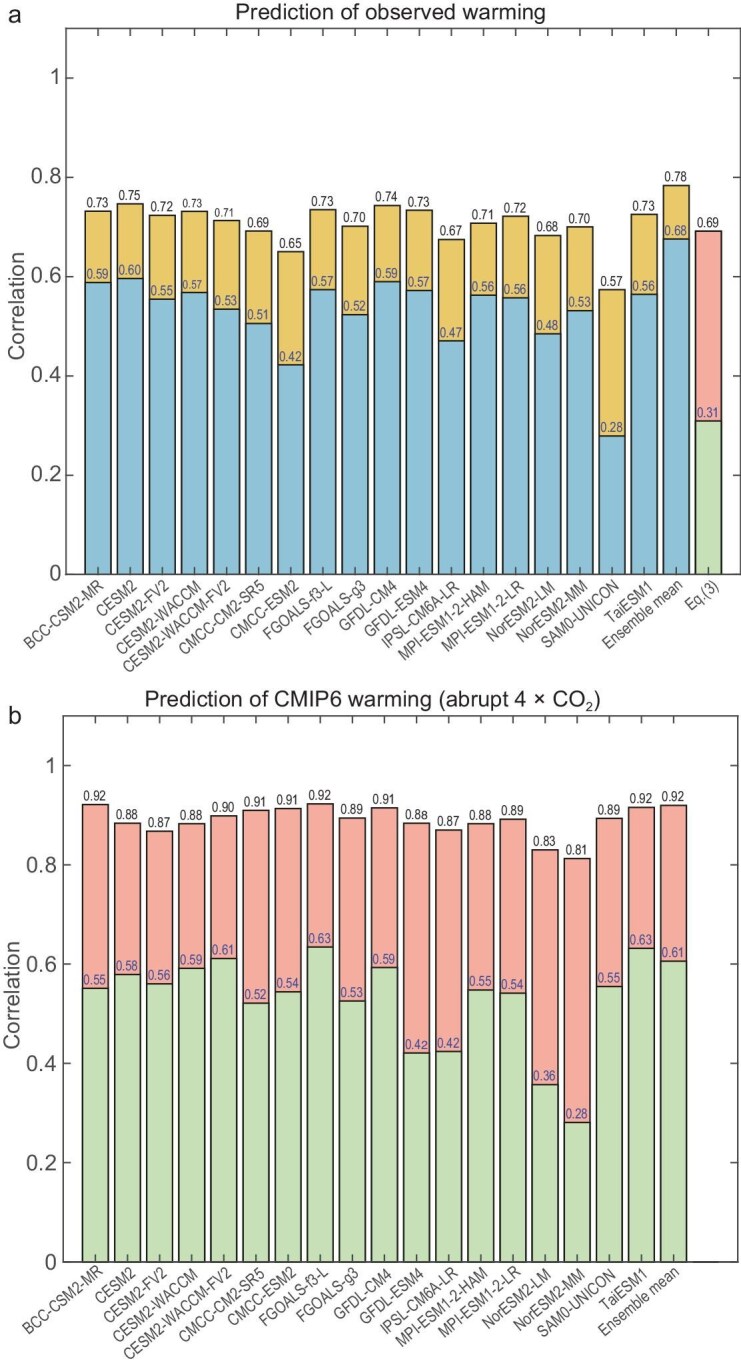
Map-correlation skill of warming predictions. (a) Predictions by individual CMIP6 historical simulations against the observed warming, (b) our predictions of warming in response to the quadrupling of CO_2_ against individual CMIP6 warming projections under the abrupt 4 × CO_2_ scenario. To ensure an equal-area representation of all grid points when calculating correlations, we divide Earth's surface into 2000 equal-area grid points. The numbers at the top of the bars correspond to the map-correlation skills including their global mean values, as given in Equation (6a), while the numbers below correspond to the map-correlation skills with their global mean values removed, as given in Equation (6b).

To further compare our prediction skill with those of CMIP6, we first obtain map-correlation skills of the CMIP6 historical simulations for the observed warming. It is seen from Fig. 6a that the map-correlation skills of individual CMIP6 historical simulations with the observed warming obtained by using Equation (6a) range from 0.57 to 0.75, all of which are below the skill of their ensemble-mean simulation (0.78). Despite only considering the observed changes in the CO_2_ concentration level, the map-correlation skill of our warming predictions against the observed warming is also as high as 0.69. The map-correlation skills of our predictions for warming in response to the quadrupling of CO_2_ with their original warming projections under the abrupt 4 × CO_2_ scenario range from 0.81 to 0.91 (Fig. 6b). Such high positive map-correlation skills of our warming predictions are noticeably better than those of climate models for the observed warming (Fig. 6a). Recall that our predictions of the CMIP6 warming projections utilize the same external forcing information as that used for the CMIP6 warming projections (i.e. the quadrupling of CO_2_). Similarly, CMIP6 historical simulations for the observed warming include all observed external forcings, such as solar variability, volcanic aerosols and anthropogenic greenhouse gases and aerosols, as well as internal climate variability [48]. Therefore, our prediction skill of warming is highly comparable to those of CMIP6 when the external forcing information is adequately considered.

However, our predictions do not seem to capture the spatial variability of the observed warming very well, as indicated by a low map-correlation skill (0.31) when the global mean warming is removed according to Equation (6b) in Supplementary Text 2. This is considerably lower than the counterparts that are derived from the historical simulations of CMIP6 models, which are all in the range of 0.42–0.6 except for one model with a skill of 0.28. The underperformance of our prediction for the spatial variability of the observed surface temperature changes can be largely attributed to the exclusion of volcanic/anthropogenic aerosols and internal climate variability in our predictions. This is evident from the comparable skill of our predictions for the spatial variability of warming projections that were made by individual climate models that were forced solely by CO_2_ changes, which mostly ranges from 0.42 to 0.61, except for two predictions with map-correlation skills of 0.36 and 0.28, when the global means are removed. Therefore, our prediction skill for the spatial variability of global warming is also highly comparable to that of CMIP6 when the external forcing information is adequately considered.

## DISCUSSION

In this study, we devise a novel principle-based framework for predicting global warming in response to external forcings imposed on Earth's climate state from the climate mean state. Our predictions only consider the temperature and non-temperature feedback in atmosphere–surface columns, excluding the oceanic response to external energy perturbations. Amplification through the EGK of the temperature feedback results in a 6.6-fold increase in the global mean of CO_2_-induced energy perturbations at the surface, while non-temperature feedback contributes to an additional 2-fold amplification. By accurately accounting for temperature- and non-temperature-feedback amplifications based on the climate mean state, we can predict global warming under any CO_2_-increasing scenario, including observed scenarios, without the need to run climate models.

Unlike the temperature kernel, which necessitates prior information on air temperature changes to calculate the amplification to external energy perturbations, as is commonly done in the literature (see e.g. Zeppetello *et al.* [49]), the EGK of the temperature feedback allows direct calculations of this amplification without the need to run climate models. The key assumption that is invoked in the principle-based framework is that the multiplication factor of the external energy perturbations at the surface by non-temperature feedback in a perturbed climate state, which is referred to as *A*_NT_, is equivalent to the energy amplification by the downward LW radiation that is emitted from the atmosphere to surface absorption of the solar energy in the climate mean state. Essentially, we utilize the *A*_NT_ that is derived from the climate mean state to estimate how non-temperature feedback amplifies the external energy perturbations at the surface in a perturbed climate state, eliminating the need to run models. We have demonstrated a strong correlation (∼0.9) between the estimated *A*_NT_ that is derived from climate mean states and the *A*_NT_ that is diagnosed from actual energy perturbations due to non-temperature feedback. This validation supports our approach of estimating the *A*_NT_ from climate mean states without the need to run climate models.

We assess the prediction capability of the principle-based framework under different configurations, including the observed warming and global-warming projections by 18 individual CMIP6 models under both the abrupt quadrupling of increasing CO_2_ and the 1% annual increase in CO_2_ scenarios. Our predictions closely match the observed global mean warming from 1980–2000 to 2000–2020 (0.403 K predicted vs. 0.414 K observed). In comparison, the global mean warming that was captured by the ensemble-mean CMIP6 historical simulation is 0.516 K, with a median global mean warming of 0.526 K. The mean absolute difference between our predictions and the CMIP6 projections is <20% of the ensemble-mean global-warming projection, which is less than the counterpart CMIP6 historical simulations for the observed global warming (∼57%). The global mean absolute error of our prediction for the observed warming (∼0.30 K) is comparable to those of CMIP6 historical simulations (0.30−0.46 K). The map-correlation skill of our reproduction of the CMIP6 warming projections ranges from 0.81 to 0.91; they are mostly between 0.4 and 0.6, even with the global mean warming removed. This level of correlation is highly comparable to that of individual CMIP6 models for the observed warming. In comparison with the CMIP6 historical simulations, the main error in our predictions, excluding the oceanic response to CO_2_ forcing, is the systematic underestimation of warming over land.

Our results indicate that the global mean difference between the observed trends from 1980–2000 to 2000–2020 and our prediction is very close to zero. This implies that the residual effects of the negative phase of the PDO that caused the global-warming hiatus in 2000–2010, along with the strengthening response of oceanic upwelling over the Southern Oceans and the weakening of the AMOC, made minimal contributions to the overall global warming that was observed from 1980–2000 to 2000–2020. Therefore, the CO_2_ increase, and not natural variability, was the main factor that was responsible for the observed warming trends from 1980–2000 to 2000–2020. Nearly 80% of the CMIP6 global mean warming projections are attributed to the direct response to the abrupt 4 × CO_2_ amplification through temperature- and non-temperature-feedback processes within atmosphere–surface columns. The remaining 20% are attributed to the oceanic response. Our identification of anthropogenic forcing as the primary cause of the observed global warming is principle-based, marking it as the first determination of the cause of global warming without reliance on climate models and statistical analysis.

Aside from excluding the oceanic response to CO_2_ forcing, the primary limitation of the new framework for predicting global warming lies in the approximation of the additional multiplication factor from non-temperature feedback (i.e. *A*_NT_) for the amplified surface external energy perturbations by temperature feedback. It is assumed that the *A*_NT_ can be inferred from the energy amplification by the downward LW radiation that is emitted from the atmosphere to surface absorption of the solar energy in the climate mean state. This approximation may be inaccurate unless the amplified surface external energy perturbations by temperature feedback exhibit a similar global scale pattern to the surface absorption of the solar energy in the climate mean state. As illustrated in Fig. S11, the amplified CO_2_-induced energy perturbations at the surface do exhibit a similar global scale pattern to the surface absorption of the solar energy in the climate mean state. Likewise, the *A*_NT_ that is derived from the mean climate state shows a comparable pattern to the actual *A*_NT_ that is derived from perturbed climate simulations. This explains why our predictions demonstrate comparable skill to those of climate models for CO_2_-induced global warming. However, our prediction may not be skillful when external forcing has a distinct spatial pattern that bears little resemblance to the surface absorption of solar energy in the climate mean state. For instance, in cases of negative external forcing due to decreasing CO_2_ or solar radiation management geoengineering [50], the new prediction framework would not accurately predict the resultant global cooling. This is because the mean state energy balance equation lacks information regarding the multiplication factor from non-temperature feedback in response to negative surface energy perturbations. Moreover, the new prediction framework can only predict the equilibrium response of the surface temperature to external forcing. As a result, it cannot capture the internal variability response to external forcing and potential tipping points that could trigger abrupt climate changes due to non-linear interactions of temperature and non-temperature feedback.

## Supplementary Material

nwae442_Supplemental_File

## Data Availability

The data used for this study can be downloaded from: ERA5 monthly mean 3D data at https://cds.climate.copernicus.eu/cdsapp#!/dataset/reanalysis-era5-pressure-levels-monthly-means?tab=form, ERA5 monthly mean surface data at https://cds.climate.copernicus.eu/cdsapp#!/dataset/reanalysis-era5-single-levels-monthly-means?tab=form and the CMIP6 model data at https://esgf-node.llnl.gov/search/cmip6/.

## CODE AVAILABILITY

The FORTRAN codes for running the radiation transfer model and calculating the EGK and external radiative forcing, plus the MATLAB scripts for calculating our global-warming predictions and plotting the results, can be available upon request.
