# Supplementary material for: Principle-based adept predictions of global warming from climate mean states
Source: Natl Sci Rev. 2024 Nov 30;12(2):nwae442. doi: 10.1093/nsr/nwae442 (PMC11968647; doi:10.1093/nsr/nwae442)

**Supplementary Data**

**for**

Principles-Based Adept Predictions of Global Warming from Climate Mean States

Ming Cai^1^*, Xiaoming Hu^2,3^, Jie Sun^1^, Yongyun Hu^4^, Guosheng Liu^1^, Zhaohua Wu^1^, Feng Ding^4^, and Wanying Kang^5^

*^1^ Department of Earth, Ocean, and Atmosphere Science, Florida State University; Tallahassee, Florida, 32306, USA.*

*^2^ School of Atmospheric Sciences, Sun Yat-sen University and Southern Marine Science and Engineering Guangdong Laboratory (Zhuhai); Zhuhai, 519082,*

*P. R. China.*

*^3^ Guangdong Province Key Laboratory for Climate Change and Natural Disaster Studies; Zhuhai, 519082, P. R. China.*

^4^ Department of Atmospheric and Oceanic Sciences, Peking University, Beijing, 100871 P. R. China

^5^ Department of Earth, Atmospheric, and Planetary Sciences, Massachusetts Institute of Technology, Boston, Massachusetts, 02139, USA

*Corresponding author: Ming Cai; **Email:**  [mcai@fsu.edu](mailto:mcai@fsu.edu)

**This PDF file includes:**

- **Supplementary Text 1**: **Methods**
- **Supplementary Text 2**: **Estimating** $\mathbf{A}_{\mathbf{NT}}$ **from the climate mean surface energy**

**balance and validations**

- **Supplementary Figures** **S1 to S11**
- **Supplementary Table S1**

**Supplementary Text 1: Methods**

**Reanalysis dataset**

The monthly mean data of the European Centre for Medium-Range Weather Forecasts (ERA5) reanalysis [29] covering the period of 1980-2000 are used to construct climate mean states of three-dimensional (3D) temperature, specific humidity, clouds, and ozone fields and two-dimensional (2D) surface albedo, surface pressure, and surface temperature fields. The ERA5 reanalysis is archived at the Climate Data Store (https://cds.climate.copernicus.eu/cdsapp#!/home). The horizontal resolution of the ERA5 reanalysis dataset is 1.5$^{\circ}$×1.5$^{\circ}$ in longitude and latitude, and there is a total of 37 pressure levels plus the surface level. Only data above the surface pressure level are used in all calculations.

In addition, we use the monthly mean surface temperature data derived from ERA5 covering the period of 2000–2020 to obtain the 2000–2020 mean surface temperature. The difference between the 2000–2020 and 1980–2020 mean surface temperatures corresponds to the observed warming from 1980–2020 to 2000–2020, which is used to validate our prediction of the observed warming.

**CMIP6 datasets**

The CMIP6 climate simulation output files are stored at the ESGF P2P (Earth System Grid Federation Peer-to-Peer) distributed data archive and can be downloaded at <https://esgf-node.llnl.gov/search/cmip6/>. The output fields of all CMIP6 models are given at 17 pressure levels plus the surface level. Only data above the surface pressure level are used in all calculations. Information about the horizontal resolution of each CIMP6 model is provided in Table S1.

The monthly mean data derived from preindustrial simulations of 18 CIMP6 (Coupled Model Intercomparison Project Phase 6) models [40] covering the period of 1900-1910 are used to construct climate mean states of 3D temperature, specific humidity, clouds, and ozone fields and 2D surface albedo, surface pressure, and surface temperature for each of the 18 CMIP6 models. By applying the ensemble average across the 18 CMIP6 models, we obtained the ensemble-mean preindustrial climate state.

We have downloaded monthly mean surface temperature data simulated by these models for two specific scenarios: (i) the abrupt quadrupling of CO_2_ (4×CO_2_) scenario covering the period from model year 130 to 140 and (ii) the 1% annual CO_2_-increasing scenario covering the period from model year 1 to 150. The global warming projections of the CMIP6 models under the abrupt 4×CO_2_ scenario are defined as the difference between the mean surface temperature for the period of 130-140 years and the preindustrial mean surface temperature (1900-1910). The difference between the mean surface temperature for the period of 70-80 years under the 1% annual CO_2_ increase scenario and the preindustrial mean surface temperature corresponds to the transient warming response at the 2×CO_2_ level, whereas the difference between the mean surface temperature for the period of 140-150 years and the preindustrial mean surface temperature is the transient warming projection at the 4×CO_2_ level. By applying the ensemble average across the 18 CMIP6 models, we obtained the ensemble-mean global warming projections.

The predictions of individual CMIP6 models for the observed warming are derived from the climate mean surface temperature differences of their historical simulations between 2000-2014 and 1980-1994. The map correlation skills shown in Fig. 5A are obtained against the climate mean surface temperature differences of ERA5 between 2000-2014 and 1980-1994 to match the historical simulation records.

Besides the surface temperature field, we have also downloaded 3D monthly mean air temperature, specific humidity, clouds, and ozone fields, and 2D monthly mean surface albedo and surface pressure fields from the perturbed climate simulations of CMIP6’s under the abrupt quadrupling of CO_2_ (4×CO_2_) scenario covering the period from model year 130 to 140. The additional fields derived from the perturbed climate simulations are considered only for diagnosing actual energy perturbations due to non-temperature feedbacks, which are then used to validate the multiplication factor by the non-temperature feedbacks estimated from mean climate states.

***Procedures for predicting global warmings from climate mean states***

Global warming predictions from climate states require running a radiative transfer model multiple times at each horizontal grid point with input data consisting of the grid point’s climate mean vertical profiles of temperature, specific humidity, cloud fractional area, liquid cloud content, ice cloud content, and ozone, plus solar energy input at the top of atmosphere, surface albedo, surface pressure, and surface temperature with a specific CO_2_ concentration level. The radiative transfer model used in this study is the Fu-Liou radiation transfer model [27,28]. The output data from radiative transfer model calculations, all in units of Wm⁻², include shortwave (SW) heating rates and longwave (LW) cooling rates within each layer, as well as both downward and upward SW and LW fluxes across each layer.

1. *The surface value of the nontemperature feedback kernel (*$A_{NT}$).

The first set of radiative transfer model calculations is made with the standard CO_2_ concentration level, specifically 352.2 PPM for the observed 1980-2000 climate mean state and 285 PPM for the preindustrial climate state of CMIP6 simulations. For easy reference, we refer to the output data of the first set of radiative transfer model calculations as the baseline calculation. Plugging the baseline downward and upward SW and downward LW fluxes at the surface into Eq. (4) yields the estimation of the surface value of the nontemperature feedback kernel (i.e., $A_{NT}$).

1. *Energy gain kernel (G_i,j_)*

To obtain the EGK, we first obtain the Planck feedback matrix, ($\frac{\partial R_{i}}{\partial T_{j}})$, in units of $Wm^{-2}K^{-1}$. Following [25,26], we run the radiative transfer model with the standard CO_2_ concentration level and otherwise identical time mean state except that the temperature at the *j*-th layer is warmed by 1 K. The difference between the resultant LW cooling rates and those obtained from the baseline calculation is the vertical profile of the radiative cooling rates due to 1-K warming at the *j*-th layer, corresponding to the *j*-th column of ($\frac{\partial R_{i}}{\partial T_{j}})$. Repeating the same for all vertical layers yields the full matrix of ($\frac{\partial R_{i}}{\partial T_{j}})$. We obtain $(G_{i,j})$ according to

${(G}_{i,j})=$ ${(\frac{\partial R_{i}}{\partial T_{i}})\left( \frac{\partial R_{i}}{\partial T_{j}} \right)}^{-1}$ (5)

where $(\frac{\partial R_{i}}{\partial T_{i}})$ is the diagonal matrix of $\left( \frac{\partial R_{i}}{\partial T_{j}} \right)$ and $\left( \frac{\partial R_{i}}{\partial T_{j}} \right)^{-1}$ is the inverse of $\left( \frac{\partial R_{i}}{\partial T_{j}} \right)$.

1. *Energy perturbations due to changes in CO_2_* ($\Delta F_{j}^{(EXT)}$).

To obtain the vertical profile of energy perturbations due to changes in CO_2,_ we run the radiative transfer model under the same climate mean state as the baseline calculation but using different values of CO_2_ concentration levels, namely, 385 PPM for the 2000–2020 observational period, 1,140 PPM for the abrupt 4×CO_2_ scenario, or 285×(1+0.01)^year^ PPM for the 1% annual CO_2_ increase scenario. The difference between the resultant vertical profile of net radiative heating rates (SW heating rates minus LW cooling rates) and its counterpart derived from the baseline calculation corresponds to the vertical profile of energy perturbations due to changes in CO_2_, namely ($\Delta F_{j}^{(EXT)}$).

1. *Surface temperature change in response to CO_2_-induced energy perturbations (*$\Delta T_{s}$*)*

As explained in the main text, the surface temperature in response to energy perturbations due to changes in CO_2_ is determined by requiring the total energy perturbations at the surface due to changes in CO_2_, which is equal to the product of $A_{NT}$ and $\sum_{j} G_{s,j}\Delta F_{j}^{\left( EXT \right)}$, to be balanced by thermal energy emission perturbation due to surface warming, which is equal to $4\sigma\bar{T}_{s}^{3}\Delta T_{s}$, where $4\sigma\bar{T}_{s}^{3}$ is the surface Stefan-Boltzmann feedback parameter.

***Metrics for skill evaluation of predictions for CMIP6 global warming projections***

We consider map correlation skill for evaluating our predictions at the grid level. To ensure an equal-area representation of all grid points when calculating correlations, we divide the Earth's surface into 2000 equal-area grid points. Two types of map correlations between variables X and Y are considered: one with their global means included when calculating map correlation and the other with their global means removed. The former is a direct measure the closeness of X to Y at the grid level and the latter measures the skill of X in capturing the spatial variability of Y. Below are the definitions of the two types of map correlation

$R1= \frac{\sum_{n=1}^{2000} X_{n}Y_{n}}{\sqrt{\sum_{n=1}^{2000} \left( X_{n} \right)^{2}}\sqrt{\sum_{n=1}^{2000} \left( Y_{n} \right)^{2}}}$ (6a)

$R2= \frac{\sum_{n=1}^{2000} (X_{n}-<X_{n}>)(Y_{n}-{<Y}_{n}>)}{\sqrt{\sum_{n=1}^{2000} \left( X_{n}-<X_{n}> \right)^{2}}\sqrt{\sum_{n=1}^{2000} \left( Y_{n}-{<Y}_{n}> \right)^{2}}}$ (6b)

where the subscript n denotes the index for grid points and < > represents the global mean operator.

We consider the normalized absolute mean difference (NMAD) to evaluate our global warming predictions from the preindustrial climate mean states of individual CMIP6 models against their global warming projections under both abrupt 4×CO_2_ and 1% annual CO_2_ increase scenarios,

$NMAD = \frac{\frac{1}{18}\sum_{m=1}^{m=18} |\Delta T_{m}-\Delta T_{m}^{CMIP6}|}{\Delta T_{ensemble-mean}^{CMIP6}}$ (7)

where m is the index for model numbers; $\Delta T_{m}$ is our global mean warming prediction from the preindustrial climate mean state of model m; $\Delta T_{m}^{CMIP6}$ is the global mean warming projection of model m; and $\Delta T_{ensemble-mean}^{CMIP6}$ is the ensemble-mean global warming projection of the CMIP6 models.

**Supplementary Text 2: Estimating** $\mathbf{A}_{\mathbf{NT}}$ **from the climate mean surface energy balance and validations**

Both the diagonal matrix of the Planck feedback matrix and the energy gain kernel in Eq. (2) can be determined from climate mean states using a standard radiative transfer model. Anthropogenic radiative forcing can also be calculated from climate mean states using the same radiative transfer model under specified CO_2_ concentration scenarios. However, the diagonal matrix, $\left( 1+\lambda_{i,i} \right)$, which corresponds to the vertical profile of the multiplication factors to the external energy input perturbations from nontemperature feedback, cannot be determined without running climate models or applying trend analysis to nontemperature feedback variables, such as water vapor, clouds, snow/ice coverage, and atmospheric energy transport. Therefore, one cannot use the perturbation energy balance equation, Eq. (2), to predict global warming in response to anthropogenic radiative forcing, despite the centrality of the energy gain kernel to the total climate feedback kernel.

To facilitate a transition of Eq. (2) from its primary diagnostic function to a predictive function for global surface warming, we closely examine its surface component, which is

$\frac{\partial R_{S}}{\partial T_{S}}\Delta T_{S}=\sum_{j} G_{s,j}(1+\lambda_{j,j})\Delta F_{j}^{\left( EXT \right)}$ (8)

where the subscript “*S*” denotes the surface layer and “*j*” denotes the individual atmospheric layer or the surface layer.

To utilize Eq. (8) as a prediction capacity for global surface warmings, we next examine the surface energy equation of mean climate states, which can be written as

$\frac{\bar{R}_{s}^{\uparrow}+\bar{Q}_{LH}+\bar{Q}_{SH}+\bar{O}_{s}}{\bar{S}_{s}^{\downarrow}-\bar{S}_{s}^{\uparrow}}$ $-1=$ $\frac{\bar{R}_{s}^{\downarrow}}{\bar{S}_{s}^{\downarrow}-\bar{S}_{s}^{\uparrow}}$ (9)

where $\bar{S}_{s}^{\downarrow}-\bar{S}_{s}^{\uparrow}$ is the climate mean solar energy absorbed at the surface; is the surface’s thermal radiative emission of climate mean states; $\bar{Q}_{LH}$ and $\bar{Q}_{SH}$ are the mean surface latent and sensible heat fluxes, respectively (positive for upward); $\bar{O}_{s}$ represents the oceanic storage and dynamic energy transport term (positive for ocean heat uptake, i.e., downward and for divergence of oceanic energy transport); and $\bar{R}_{s}^{\downarrow}$ is the climate mean downward LW flux at the surface from the atmosphere.

For a hypothetical climate system whose atmosphere does not emit/absorb LW energy, the right-hand side of Eq. (9) must be zero. For such a hypothetical climate system, the surface energy output is exactly equal to the energy input from the Sun. Therefore, positive values of the right-hand side of Eq. (9) indicate that the ratio of the energy output from the surface to the energy input from the Sun in climate mean states exceeds one, implying additional energy input to the surface from the atmosphere. This additional energy input results from the combined effects of atmospheric greenhouse effects, poleward energy transport, and vertical convections, all represented by the term in the numerator of the right-hand side of Eq. (9).

To make use of the surface energy equation of mean climate states for predictions of global surface warming, we rewrite the surface perturbation energy equation (8) symbolically as

$\frac{\partial R_{s}}{\partial T_{s}}\Delta T_{s}=A_{NT}\times\sum_{j} G_{s,j}\Delta F_{j}^{(EXT)}$ (10)

where $A_{NT}$ is defined as

$A_{NT}=\frac{\sum_{j} G_{s,j}(1+\lambda_{j,j})\Delta F_{j}^{(EXT)}}{\sum_{j} G_{s,j}\Delta F_{j}^{(EXT)}}$ (11)

Now, one would immediately see an analogy between the right-hand sides of Eqs. (9) and (10), namely, that they both represent the multiplication factor to the external energy input at the surface. The former corresponds to the multiplication factor of the solar energy input at the surface in the climate time mean state, and the latter corresponds to the multiplication factor of the total anthropogenic radiative forcing at the surface in a perturbed climate state. This analogy is rooted in the shared physical characteristics of the same climate system, one characterizing the time mean state and the other describing the perturbed climate state. On the basis of this physics-based analogy, we estimate $A_{NT}$ from the climate mean state

$A_{NT}\approx\frac{\bar{R}_{s}^{\downarrow}}{\bar{S}_{s}^{\downarrow}-\bar{S}_{s}^{\uparrow}}$ (12)

We postulate that the term $\bar{R}_{s}^{\downarrow}$ encapsulates the collective amplification effect on the climate mean surface temperature from (i) surface components of water vapor feedback and the LW portion of cloud feedback, including their amplifications by temperature feedback (i.e., through the amplification factor represented by $G_{s,s}$) and (ii) the effect of the energy gained at the surface from the amplifications by temperature feedback (i.e., through the factors represented by $G_{s,j}$) of atmospheric components of all nontemperature radiative and nonradiative feedback processes. For example, the surface downward LW radiation in the place where clouds are present would be different from the place where there are no clouds. The same argument applies to water vapor. Also there would be stronger (weaker) surface downward LW radiation in the region where horizontal transport of energy is convergent (divergent). We envision that the term $\bar{S}_{s}^{\downarrow}-\bar{S}_{s}^{\uparrow}$ encodes the effect of surface albedo feedback and the surface component of shortwave cloud feedback on the surface warmth of the climate mean state, including its spatial pattern. For example, in the polar regions, large values of surface albedo would further reduce solar energy input at the surface. For the same surface downward LW radiation, *A_NT_*  would be stronger in the regions where surface albedo is greater. In summary, *A_NT_* would have different values with or without clouds, with low-cloud or high-clouds, with more water vapor or less water vapor, over the region where surface albedo varies greatly. Also *A_NT_*  tends to have larger (smaller) values in the regions where horizontal transport of energy is convergent (divergent).

In essence, utilizing the physics-based analogy to estimate the strength of nontemperature feedback for surface warming is equivalent to assuming that the ratio of $\frac{\bar{R}_{s}^{\downarrow}}{\bar{S}_{s}^{\downarrow}-\bar{S}_{s}^{\uparrow}}$, derived from the climate mean state, applies equally for the multiplication factor of any external energy input perturbations as it does for the multiplication factor of solar energy input at the surface in the climate mean state. The surface temperature change in a perturbed climate state can be determined from the balance of surface thermal emission perturbations with amplified external energy perturbations by temperature and nontemperature feedback. The results presented in the main article, Fig. S1, and Fig. S11 indicate that the estimation of *A_NT_* from the ratio of $\frac{\bar{R}_{s}^{\downarrow}}{\bar{S}_{s}^{\downarrow}-\bar{S}_{s}^{\uparrow}}$ enables skillful predictions of global mean warming and its spatial pattern in response to anthropogenic radiative forcing.

Note that the time mean surface energy balance equation can be written in a form different from Eq. (9) depending on how to account for the time mean energy output from the surface layer. In the form of Eq. (9), the time mean energy output from the surface layer is the sum of the surface thermal emission ($\bar{R}_{s}^{\uparrow}$), surface latent and sensible heat fluxes ($\bar{Q}_{LH}$ and $\bar{Q}_{SH}$), and oceanic storage and dynamic energy transport term ($\bar{O}_{s}$). Because $\bar{R}_{s}^{\uparrow}$ and $\bar{Q}_{LH}$ are always positive at any place across the globe and $\bar{Q}_{SH}$ tends to be positive at most places, there is no ambiguity in regarding them as the time mean energy output from the surface layer. Under equilibrium conditions, the global mean of $\bar{O}_{s}$ is zero, thereby not contributing to the global mean energy output from the surface. However, locally, $\bar{O}_{s}$ is positive when energy is removed from the surface layer to the deep ocean (oceanic heat uptake) or transported away to other locations by oceanic energy transport and negative when energy is returned to the surface layer from the deep ocean (oceanic heat release) or transported from other locations by oceanic energy transport. The spatial variation in the polarity of the term $\bar{O}_{s}$ results in ambiguity regarding its role in maintaining the temporal mean surface energy balance locally. Specifically, there are at least two alternative ways to incorporate the term $\bar{O}_{s}$, in addition to Eq. (9). One is to account for the term $\bar{O}_{s}$ as part of the amplification to the time mean energy input term (i.e., $\bar{S}_{s}^{\downarrow}-\bar{S}_{s}^{\uparrow}$), which leads to

$\frac{\bar{R}_{s}^{\uparrow}+\bar{Q}_{LH}+\bar{Q}_{SH}}{\bar{S}_{s}^{\downarrow}-\bar{S}_{s}^{\uparrow}}-1=\frac{\bar{R}_{s}^{\downarrow}-\bar{O}_{s}}{\bar{S}_{s}^{\downarrow}-\bar{S}_{s}^{\uparrow}}$ (9.a)

The other alternative way is to account for the term $\bar{O}_{s}$ as part of the time mean energy input to the surface layer, which leads to

$\frac{\bar{R}_{s}^{\uparrow}+\bar{Q}_{LH}+\bar{Q}_{SH}}{\bar{S}_{s}^{\downarrow}-\bar{S}_{s}^{\uparrow}-\bar{O}_{s}}-1=$ $\frac{\bar{R}_{s}^{\downarrow}}{\bar{S}_{s}^{\downarrow}-\bar{S}_{s}^{\uparrow}-\bar{O}_{s}}$ (9.b)

The two alternative forms of the time mean surface energy balance equations lead to two alternative estimates of $A_{NT}$, in addition to Eq. (12), which are

$A_{NT}\approx\frac{\bar{R}_{s}^{\downarrow}-\bar{O}_{s}}{\bar{S}_{s}^{\downarrow}-\bar{S}_{s}^{\uparrow}}$ (12.a)

$A_{NT}\approx\frac{\bar{R}_{s}^{\downarrow}}{\bar{S}_{s}^{\downarrow}-\bar{S}_{s}^{\uparrow}-\bar{O}_{s}}$ (12.b)

Obviously, the two alternative estimates of $A_{NT}$ require obtaining information about the term $\bar{O}_{s}$, which is neither directly observable nor part of the standard output fields, unlike the other terms on the right-hand side of Eqs. (12.a) and (12.b). The term $\bar{O}_{s}$ can be indirectly inferred as the residual term of the time mean surface energy balance equation, namely,

$\bar{O}_{s}=\bar{S}_{s}^{\downarrow}-\bar{S}_{s}^{\uparrow}+\bar{R}_{s}^{\downarrow} -(\bar{R}_{s}^{\uparrow}+\bar{Q}_{LH}+\bar{Q}_{SH}$) (13)

All the terms on the right-hand side of Eq. (13) are part of the standard output fields of the CMIP6 models. We applied the term $\bar{O}_{s}$, obtained using Eq. (13) from the time mean state of the CMIP6 preindustrial simulations, to Eqs. (12.a) and (12.b), resulting in two different alternative estimates of $A_{NT}$. As indicated in Extended Data Table 1, these two alternative estimates of $A_{NT}$ show small differences from our global mean predictions for individual CMIP6 models using $A_{NT}$ estimated according to Eq. (12), although they somewhat modulate spatial warming patterns. In light of this, we only report the results obtained using the estimate of $A_{NT}$ from Eq. (12) in this study. Because the estimate of $A_{NT}$ from Eq. (12) does not require any information about $\bar{O}_{s}$, our global warming predictions are made without considering the oceanic response to anthropogenic radiative forcing effectively.

The validation of $A_{NT}$ estimated from climate mean states using Eq. (12) or Eq. (4) can be made by diagnosing vertical profiles of energy perturbations due to non-temperature feedbacks, i.e., the terms $\Delta F_{j}^{(X)}$ in Eq. (1). As we obtain external energy perturbations, we also obtain $\Delta F_{j}^{(X)}$ due to water vapor, cloud, and surface albedo feedbacks by running the radiative transfer model under the same climate mean state as the baseline calculation but using the water vapor, cloud, and surface albedo fields derived from the 2000–2020 observational period or from perturbed climate simulations. The difference between the resultant vertical profile of net radiative heating rates (SW heating rates minus LW cooling rates) and its counterpart derived from the baseline calculation corresponds to the vertical profile of energy perturbations due to changes in water vapor, cloud, and surface albedo, denoted as $\Delta F_{j}^{(WV+CLD+ALB)}$. To obtain energy perturbations due to changes in vertical and advective processes, we run the radiative transfer model using the full output fields derived from perturbed climate simulations with the corresponding CO_2_ concentration. For the equilibrium climate state, the difference between the resultant vertical profile of net radiative cooling rates (LW cooling rates minus SW heating rates) and its counterpart derived from the baseline calculation corresponds to the vertical profile of energy perturbations due to changes in convective and advective processes, denoted as $\Delta F_{j}^{(DYN)}$. Then the energy amplification kernel by non-temperature feedbacks can be obtained according to

${1+\lambda}_{j,j}=1+\frac{\Delta F_{j}^{(WV+CLD+ALB)}+\Delta F_{j}^{(DYN)}}{\Delta F_{j}^{(EXT)}}$ (14)

Substituting (14) into (11), we obtain $A_{NT}$ through diagnostic analyses of actual energy changes due to nontemperature feedbacks.

Shown in Fig. S1A is the scatter plot of *A_NT_* diagnosed from CMIP6 ensemble mean perturbed climate simulations (ordinate) versus that estimated from CMIP6 ensemble mean preindustrial climate mean state (abscissa). It is seen that *A_NT_* estimated from CMIP6 ensemble mean preindustrial climate mean state exhibits high positive correlation (~0.91) with its diagnoses from actual energy perturbations due to nontemperature feedbacks derived from CMIP6 ensemble mean perturbed climate simulations. The same map correlation analysis reveals that *A_NT_* estimated from individual CMIP6 preindustrial climate mean states are all well correlated with their counterparts diagnosed from actual energy perturbations due to nontemperature feedbacks in CMIP6 simulations for warming projections, ranging from 0.83 to 0.93 (Fig. S1C). Despite only considering the observed changes in the CO_2_ concentration level, the map correlation skill of *A_NT_* estimated from the 1980-2000 climate mean state is still well correlated (~.76) with its diagnoses from the actual energy perturbations due to nontemperature derived from the differences between 2000-2020 and 1980-2000 climate mean states.

However, *A_NT_* estimated from CMIP6 ensemble mean preindustrial climate mean state does not equally well capture the spatial variability of the actual *A_NT_* as indicated noticeably smaller map correlation skills when the global means of *A_NT_* are removed, ranging from 0.17 to 0.61. Recall that it is the product of temperature and nontemperature feedbacks’ amplifications that is actually considered in our global warming predictions, as shown in Eq. (3). Because the amplification by temperature feedback is 2-10 times greater than that by nontemperature feedbacks (Fig. S1B compared to Fig. S1A), the relatively lower map correlation skill for the spatial variability of *A_NT_* does not significantly impact the accuracy of our global warming predictions at the grid level. This is evident from much higher map correlation skills for the spatial variability of the total amplification (Fig. S1D), which range from 0.47 to 0.94, and the even higher map correlation skills when the global mean value is included in map correlation analysis (0.85 to 0.98). This explains why our prediction skill for global warming is highly comparable to that of CMIP6 when the external forcing information is adequately considered, as demonstrated in Fig. 5.

**Fig. S1**.

**
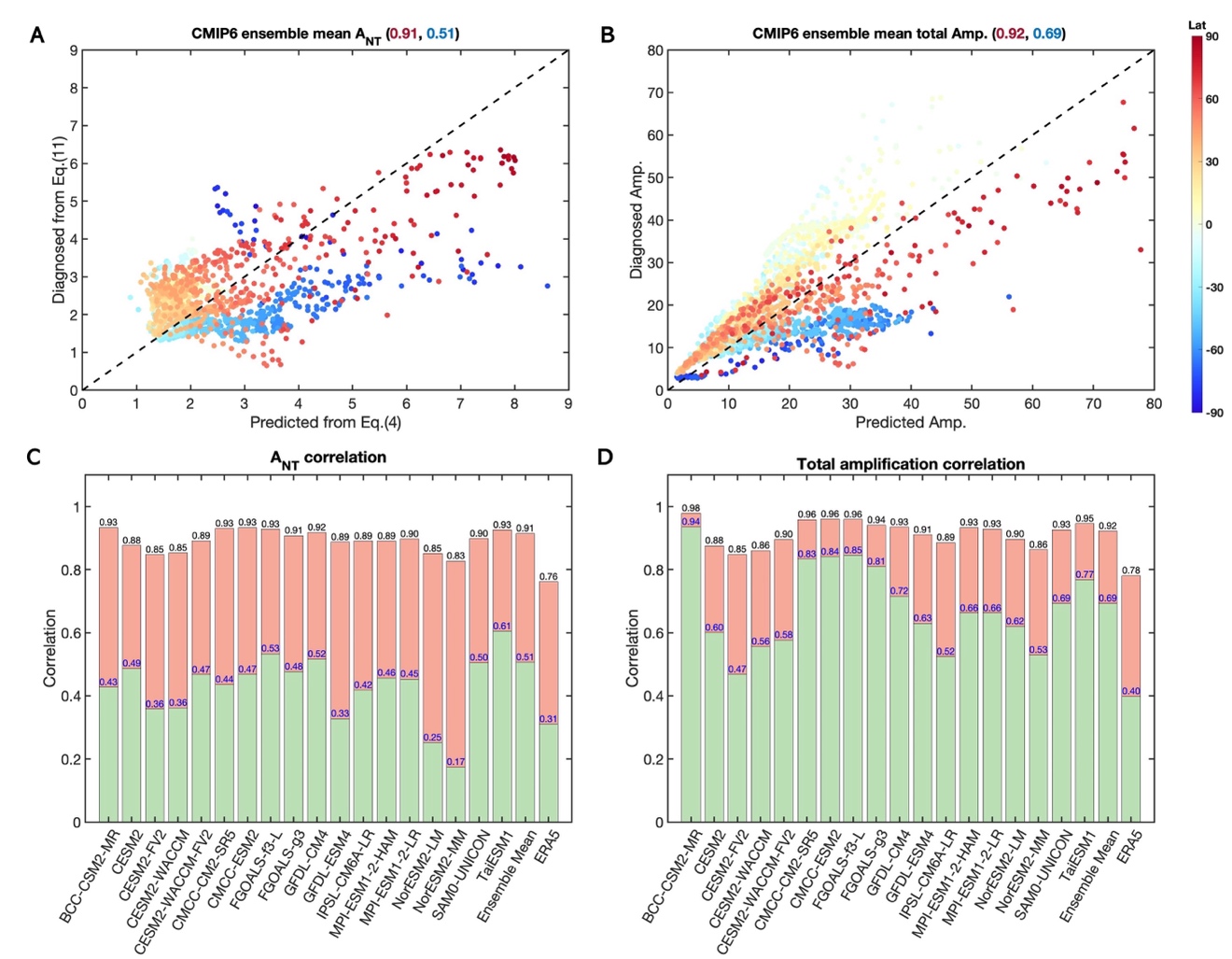
**

**Fig. S1. Validation of the multiplication factor by nontemperature feedbacks (*A_NT_*) estimated from climate mean states.** (A) Scatter plot of *A_NT_* diagnosed from CMIP6 ensemble mean perturbed climate simulations (ordinate) versus that estimated from CMIP6 ensemble mean pre-industrial climate mean state (abscissa); (B) Scatter plot of the total amplification diagnosed from CMIP6 ensemble mean perturbed climate simulations (ordinate) versus the one estimated from CMIP6 ensemble mean pre-industrial climate mean state (abscissa); (C) Map correlations of *A_NT_* estimated from individual CMIP6 historical climate mean states against *A_NT_*  diagnosed from individual CMIP6 perturbed climate simulations; and (D) Map correlations of total amplification estimated from individual CMIP6 historical climate mean states against that diagnosed from individual CMIP6 perturbed climate simulations. To ensure an equal-area representation of all grid points when calculating correlations, we divide the Earth's surface into 2000 equal-area grid points. The black numbers at the top of the bars in (C) and (D) correspond to the map correlations including their global mean values, as given in Eq. (6a), while the blue numbers below correspond to the map correlations with their global mean values removed, as given in Eq. (6b). The rightmost column bars in (C) and (D) are for correlation of amplifications derived from the observed mean state in 1980-2000 against their counterparts diagnosed from the differences between 2000-2020 and 1980-2000.

**Fig. S2**.


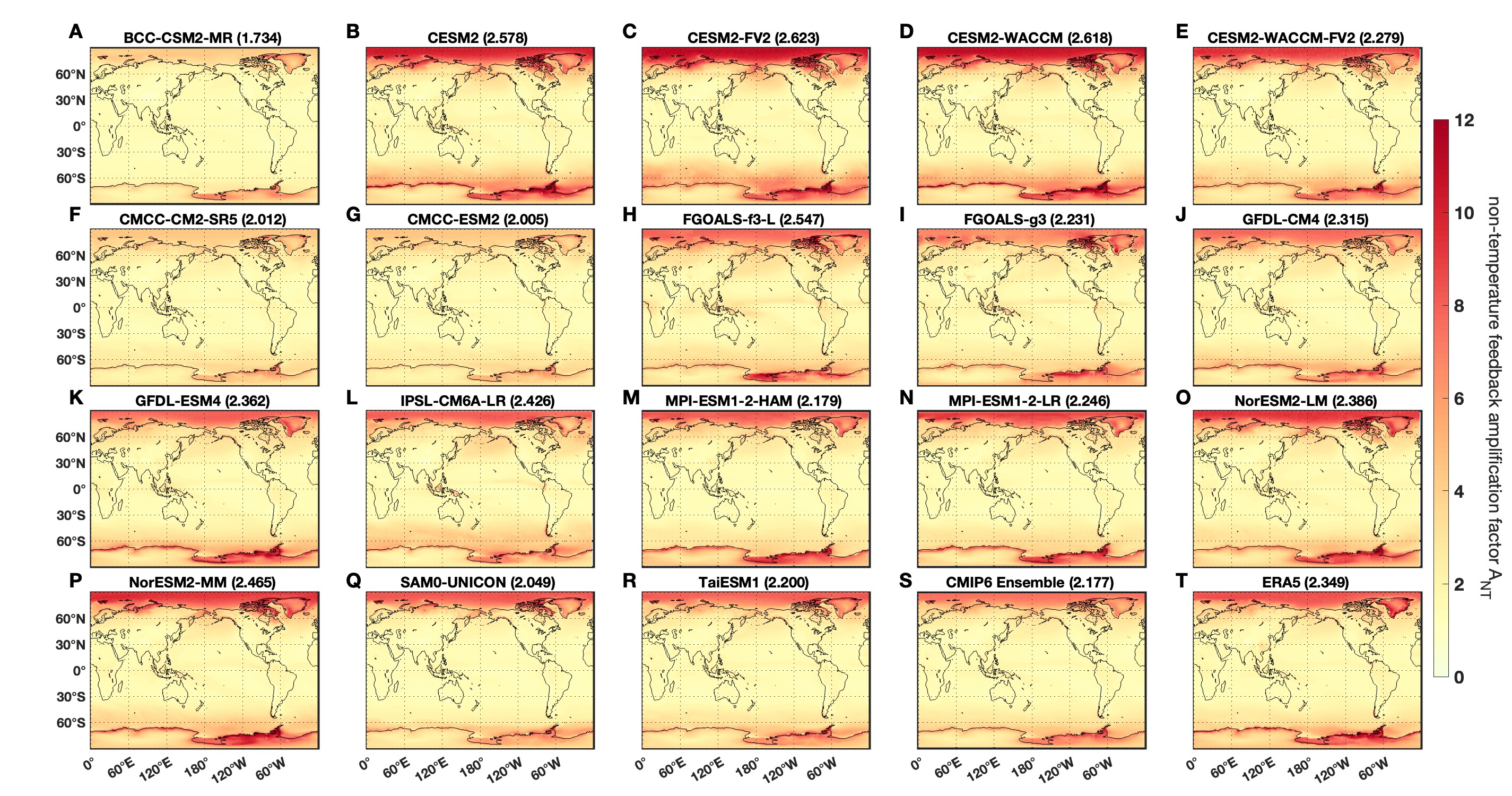


**Fig. S2. Maps of surface energy multiplication factor by nontemperature feedback.** (A)-(R) Derived from preindustrial mean states of individual CMIP6 models, (S) from the ensemble mean preindustrial mean state of CMIP6 models, and (T) from the 1980-2000 mean state of the ERA5 reanalysis. The numbers in the parentheses in the title represent the global mean values (dimensionless).

**Fig. S3**


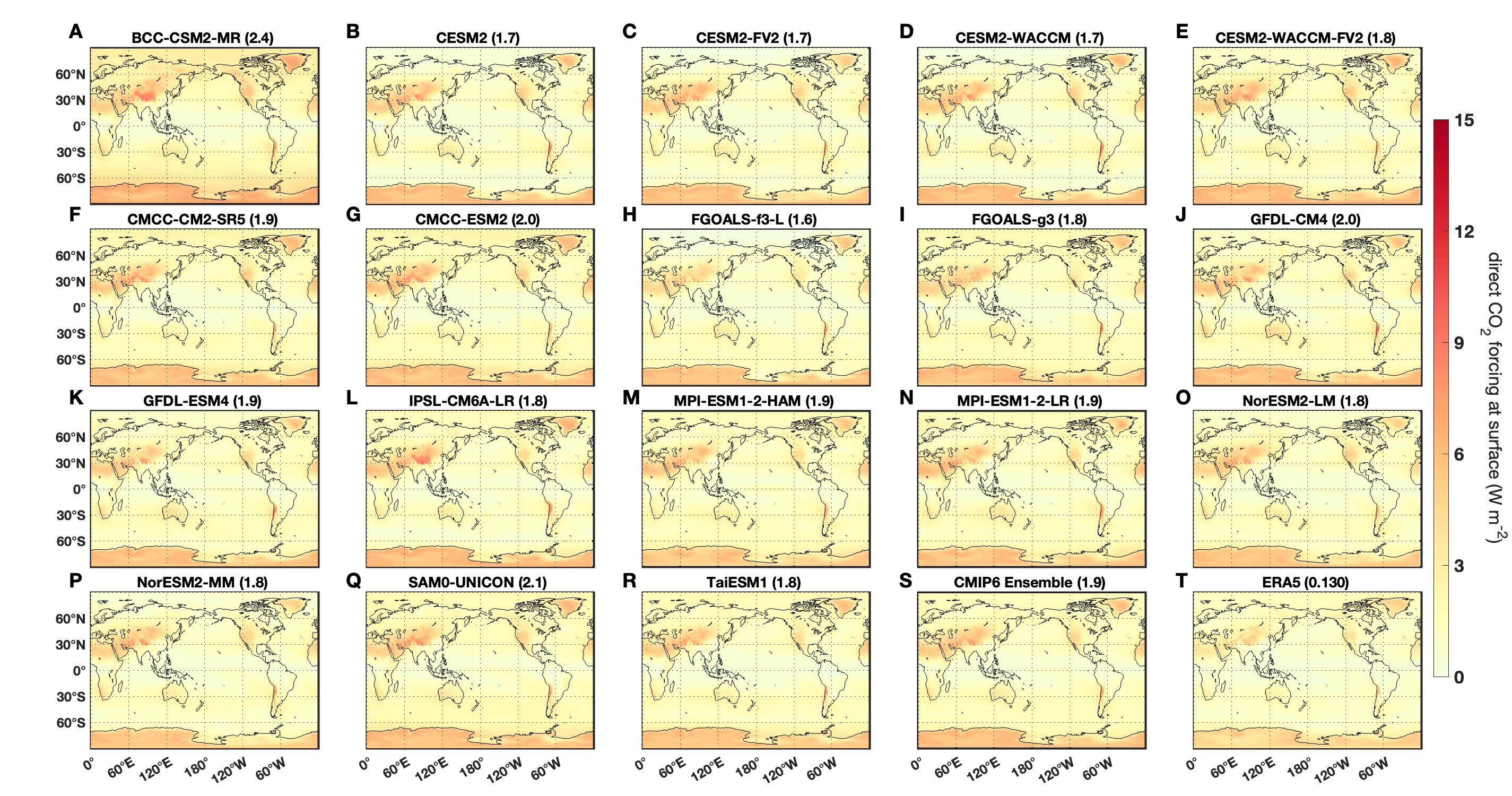


**Fig. S3. As in Figure S2 but for external energy perturbations at the surface** (Wm^−2^). Panels (A)-(S) are calculated for a CO_2_ increase from 285 PPM in the preindustrial era to 1140 PPM (the abrupt 4×CO_2_) and panel (T) is calculated from 352.2 ppm in the period of 1980-2000 to 385 PPM in the period of 2000-2020. It should be noted that the color scheme for (T) is 1/10 of the values shown in the color bar of the figure.

**Fig. S4.**


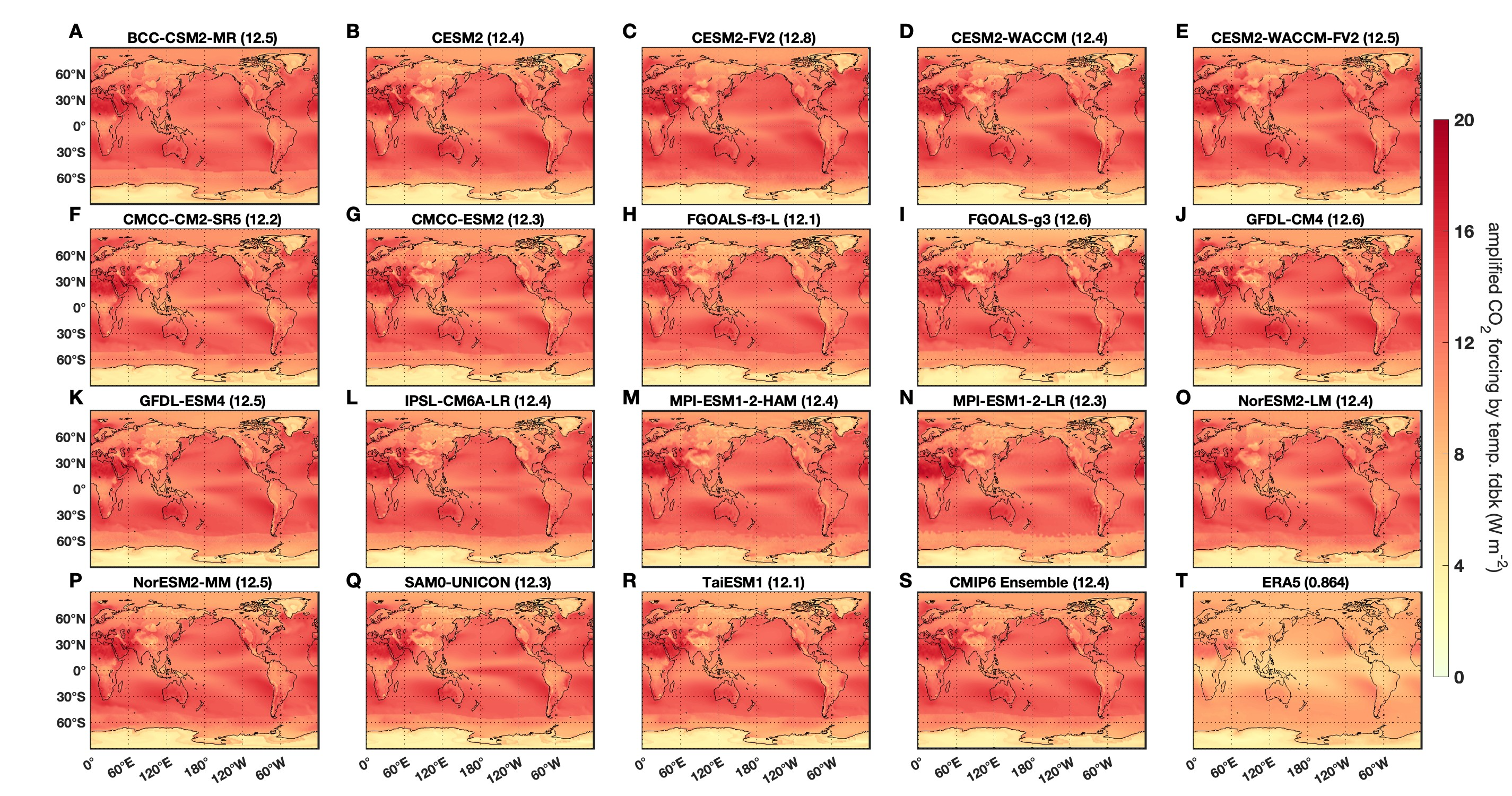


**Fig. S4. As in Figure S1 but for amplified external energy perturbations at the surface** (Wm^−2^) **through the energy gain kernel of temperature feedback**. It should be noted that the color scheme for (T) is 1/10 of the value shown in the color bar of the figure.

Fig. S5.


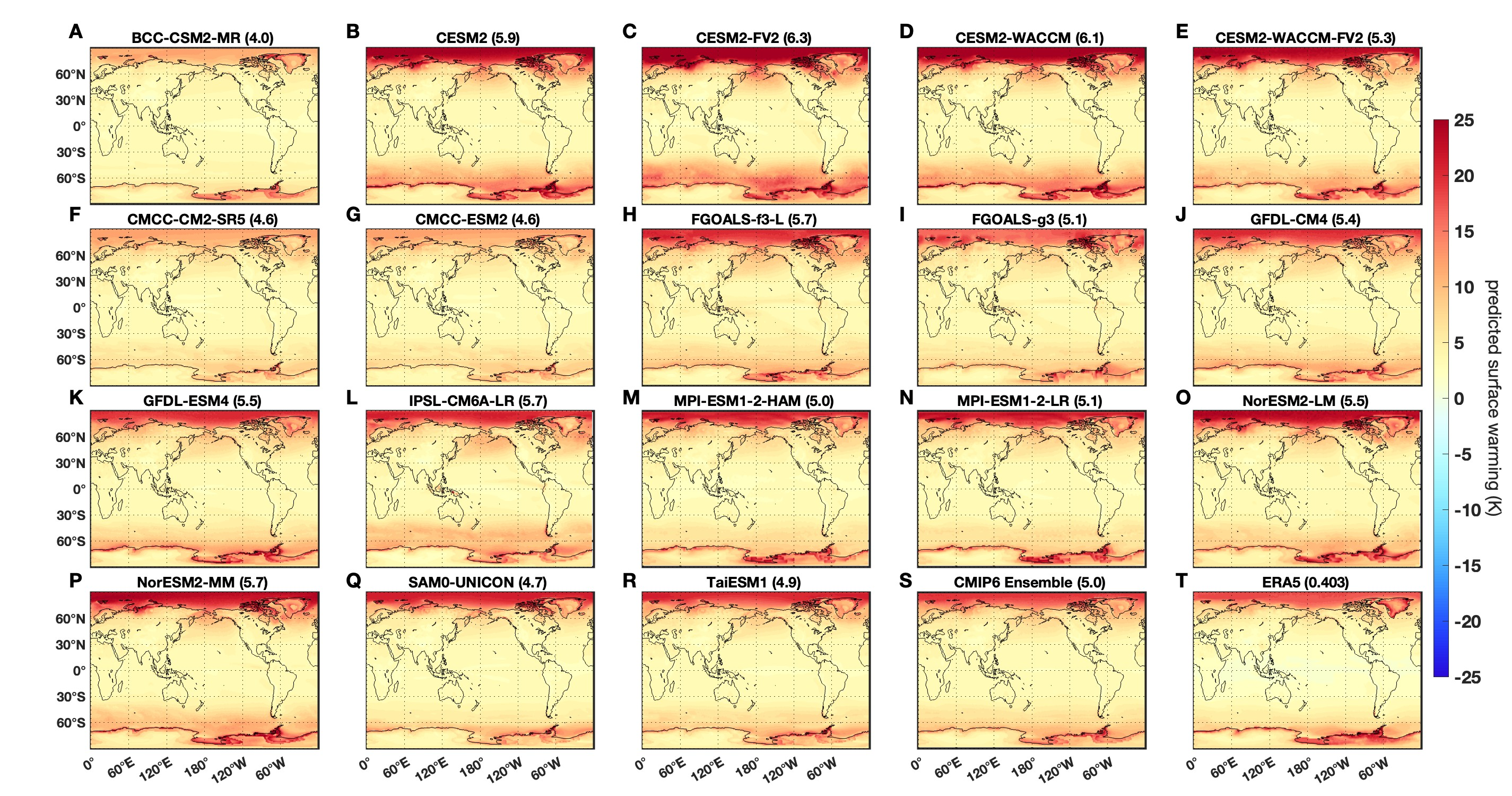


**Fig. S5. As in Figure S1 but for predicted global warming** (K). It should be noted that the color scheme for (T) is 1/10 of the value shown in the color bar of the figure.

Fig. S6.


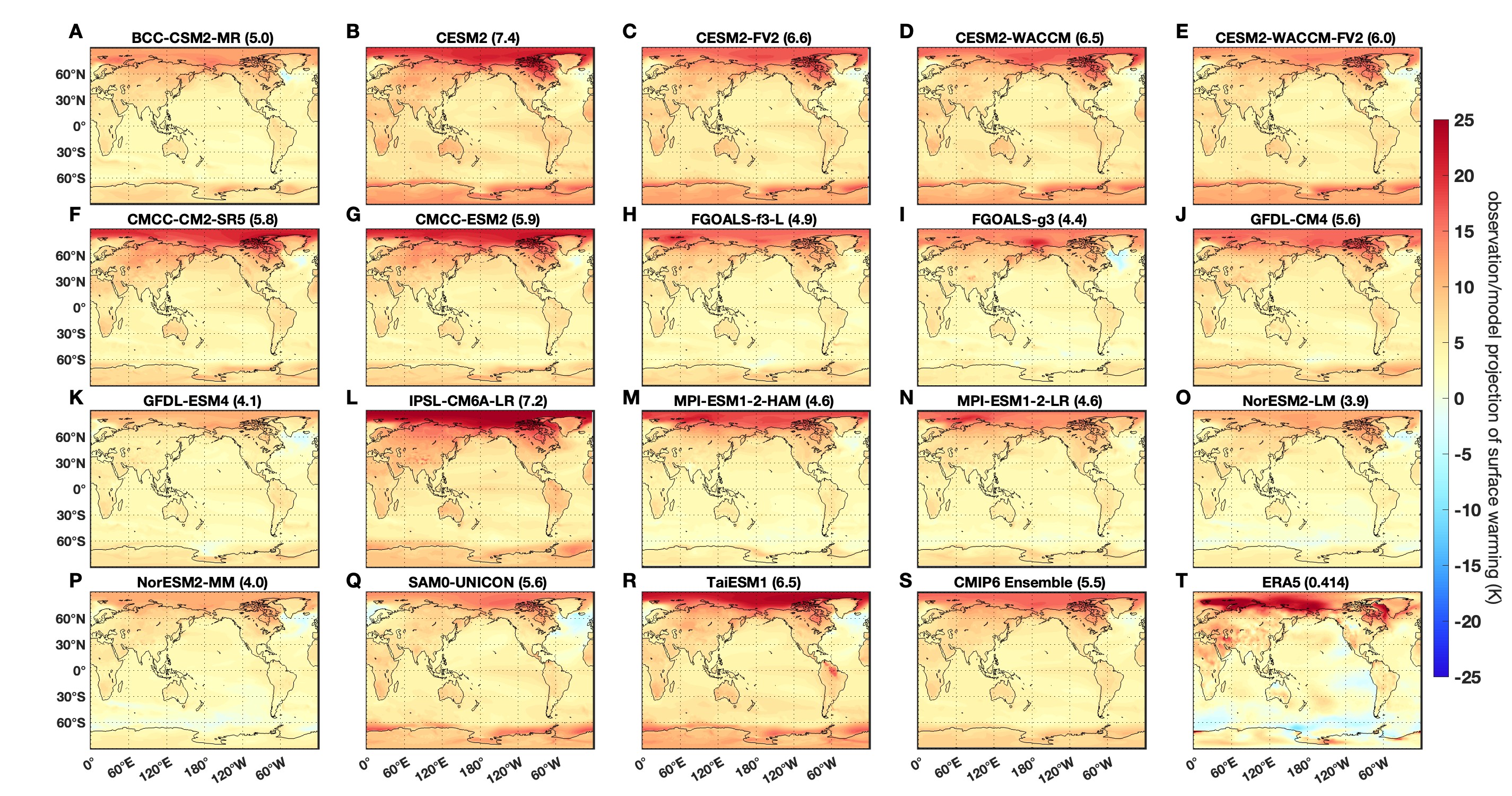


**Fig. S6. As in Figure S1 except for CMIP6 warming projections under the abrupt 4×CO_2_ scenario**. (A)-(R) Individual CMIP6 models’ warming projections, (S) CMIP6 models’ ensemble mean warming projection, and (T) the observed warming from 1980-2000 to 2000-2020. It should be noted that the color scheme for (T) is 1/10 of the value shown in the color bar of the figure.

Fig. S7.


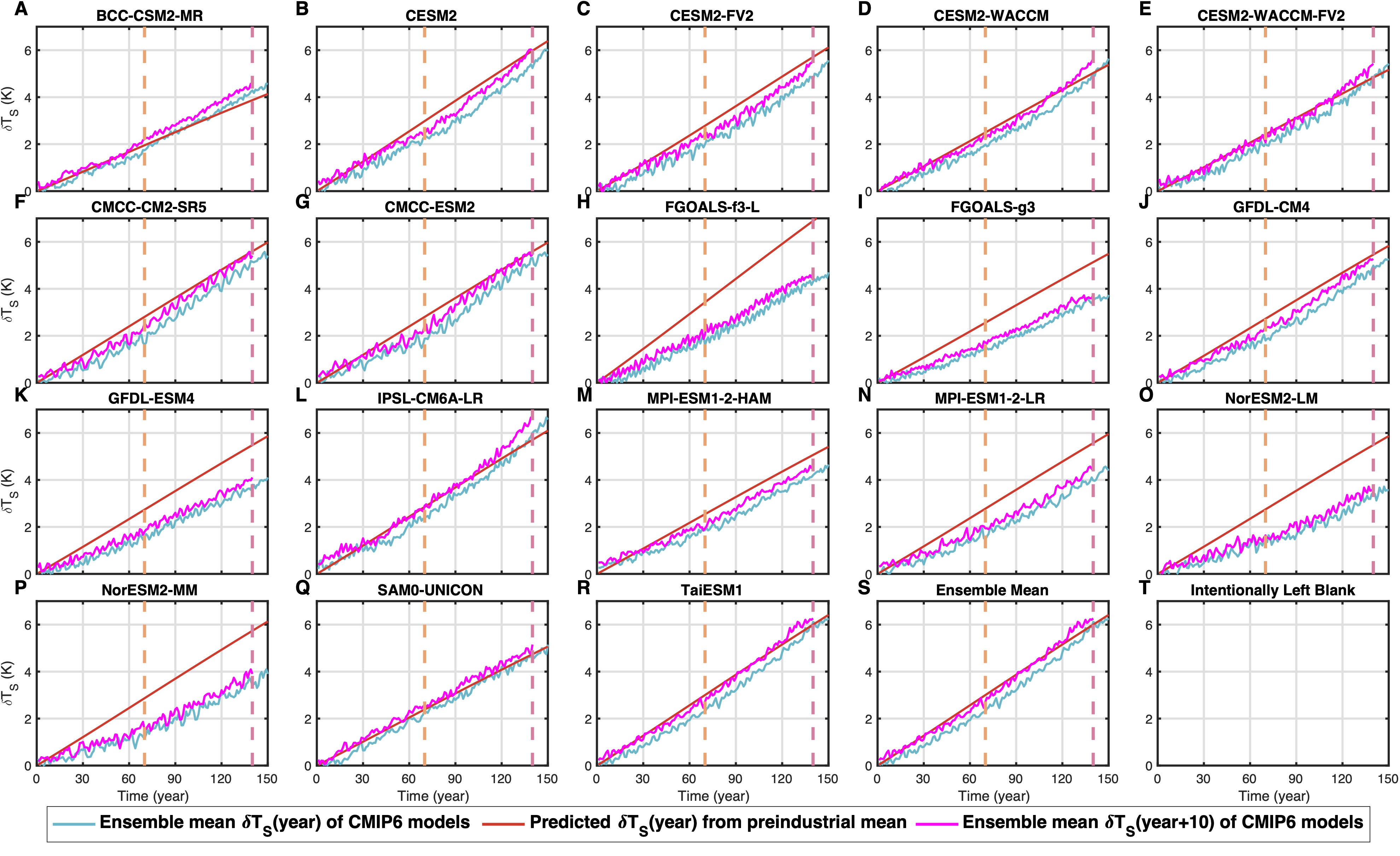


**Fig. S7. Yearly time series of global mean warming under the 1% annual CO_2_-increasing scenario.** (A)-(R) Individual CMIP6 models, and (S) the ensemble mean of CMIP6 models. Panel (T) is left blank because it is not applicable for observations. The red lines represent our predictions, and the blue lines represent the global warming projections of CMIP6 models. The magenta line is otherwise identical to the corresponding blue line except that the former is shifted 10 years ahead for a visual illustration of the delay of the continuous transient response to the 1% annual CO_2_-increase.

Fig. S8.


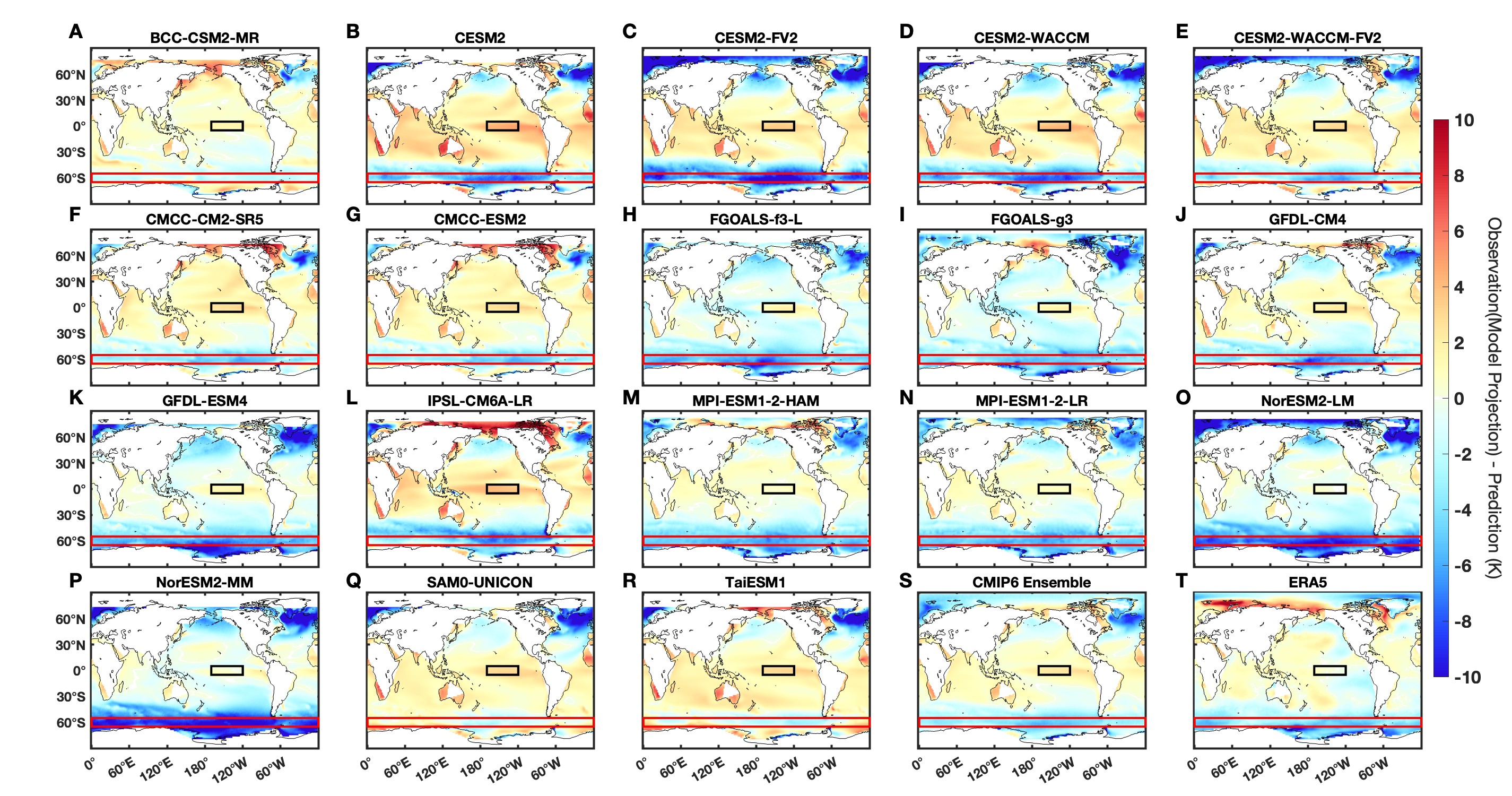


Fig. S8. As in Figure S1 but for the differences between the models/observed warming and our prediction. The differences (only ocean grid points) are obtained by subtracting the gridded data values in Figure S4 from their counterparts in Figure S5. The black box covers the area of (170°W-120°W, 5°S-5°N), and the red box covers the area of (55°S-65°S). The color scheme for (T) is 1/5 of the value shown in the color bar of the figure.

Fig. S9.


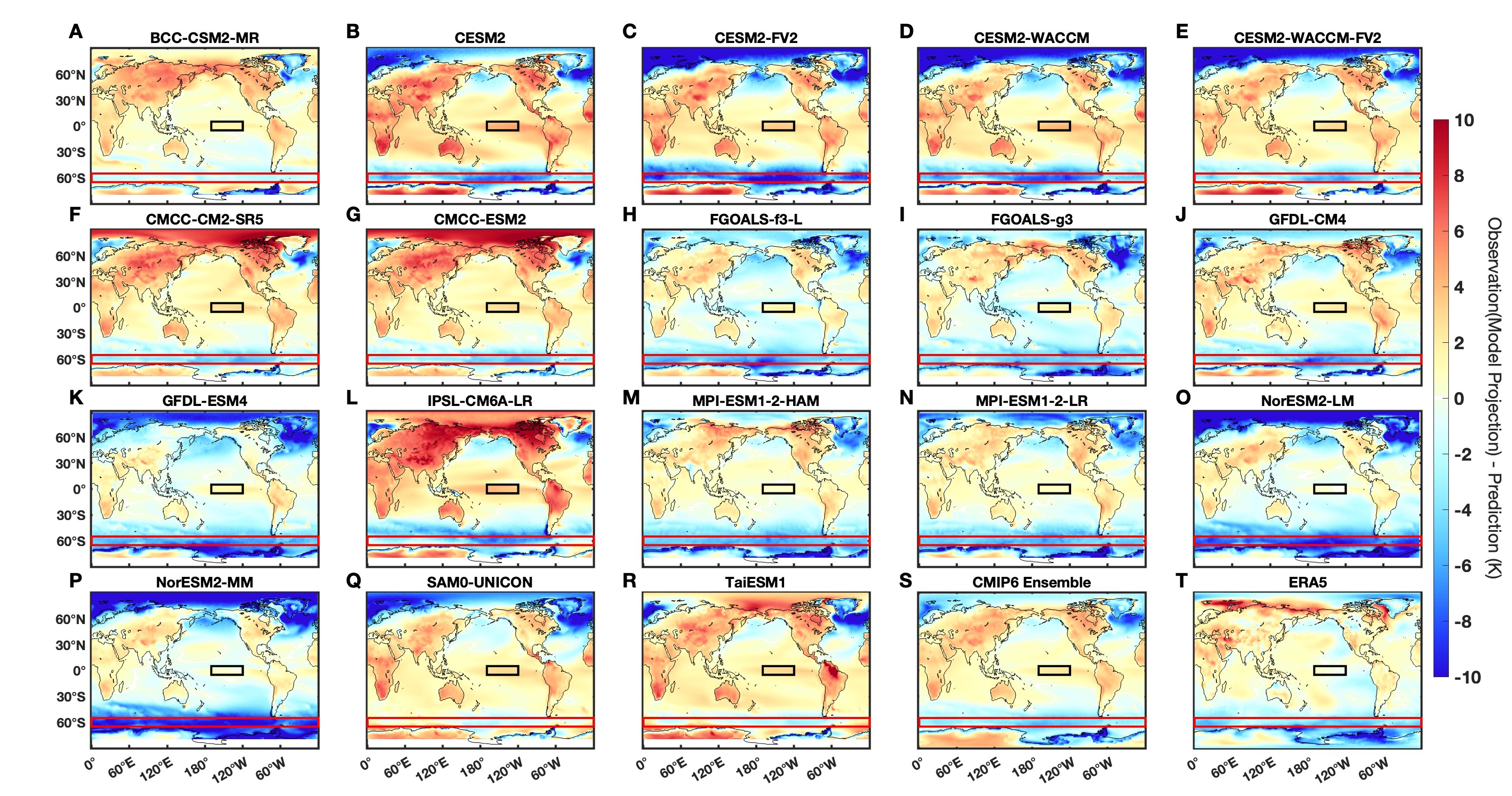


Fig. S9. As in Figure S1 but for the differences between the models/observed warming and our prediction. The differences (all grid points) are obtained by subtracting the gridded data values in Figure S4 from their counterparts in Figure S5. The black box covers the area of (170°W-120°W, 5°S-5°N), and the red box covers the area of (55°S-65°S). It should be noted that the color scheme for (T) is 1/5 of the value shown in the color bar of the figure.

Fig. S10.


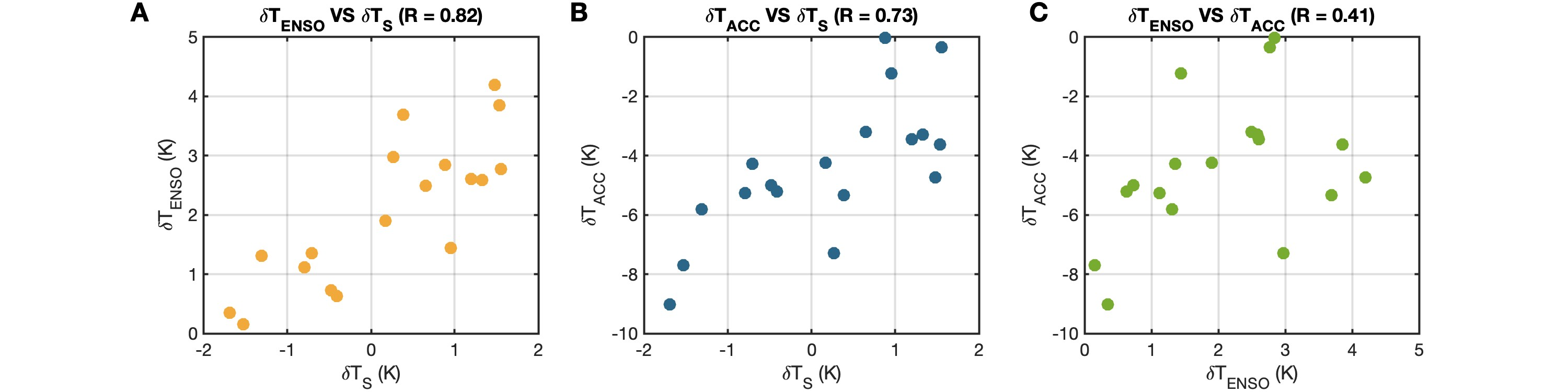


Fig. S10. Scatter plots of the difference between CMIP6’s warming projections and our predictions versus the oceanic response signal under the 4×CO_2_ scenario. (A) Global mean values of Figs. S8A-8R versus their mean values over the ENSO region (black box, 170°W-120°W, 5°S-5°N), (B) global mean values of Figs. S8A-8R versus their mean values Antarctica Circumpolar Current (ACC) region (red box, 55°S-65°S), and (C) mean values of Figs. S8A-S8R over the ENSO region versus their mean values over the ACC region.

Fig. S11


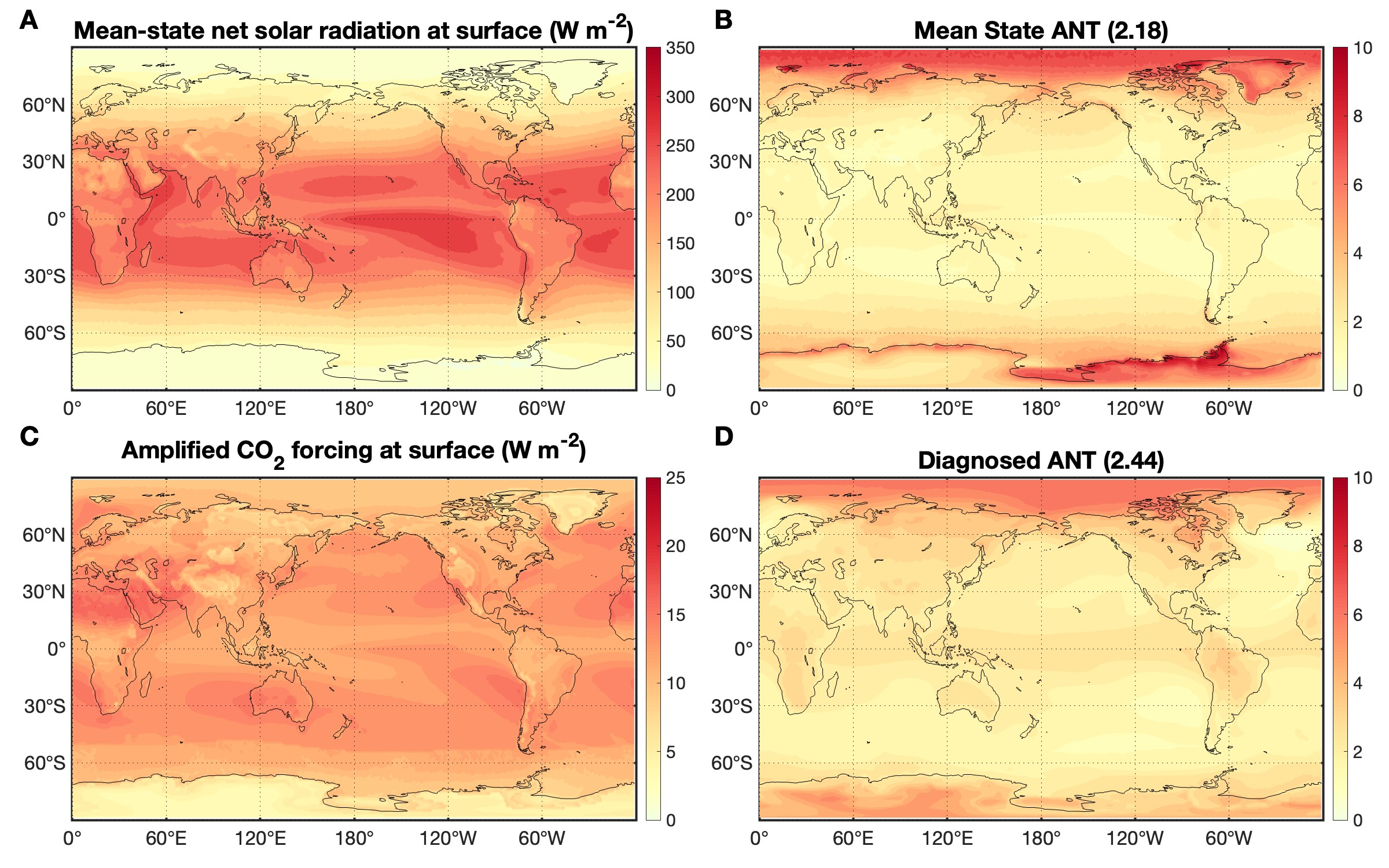


Fig. S11. Similarity in energy terms for the mean and perturbed climate states. (A) net solar radiation flux (W m^−2^) at the surface in the ensemble mean preindustrial mean state of CMIP6 models, (B) the multiplication factor (dimensionless) by non-temperature feedback derived from the ensemble mean preindustrial mean state of CMIP6 models, (C) 4×CO_2_-induced surface energy perturbations amplified by temperature feedback (W m^−2^), (D) the multiplication factor (dimensionless) by non-temperature feedback derived from CMIP6 climate projection simulations under the abrupt quadrupling of CO_2_ scenario. The numbers in the parentheses in the title of (B) and (D) represent the global mean values (dimensionless).

Table S1.

**Table S1. List of CMIP6 models, their horizontal resolutions, and their simulated global mean warming and our predictions under the 4×CO_2_ scenario.** The fourth column shows the global mean warming projections (K) of the CMIP6 models; the right three columns show our predictions (K) using three different estimates of surface energy multiplication factor by nontemperature feedback; and the fifth column shows the predictions adopted in our study. The fourth row from the bottom shows the ensemble means of the CMIP6 models, the third row the median value, and the second row their NMAD values (normalized mean absolute difference). The last row shows the observed global mean warming from 1980-2000 to 2000-2020.


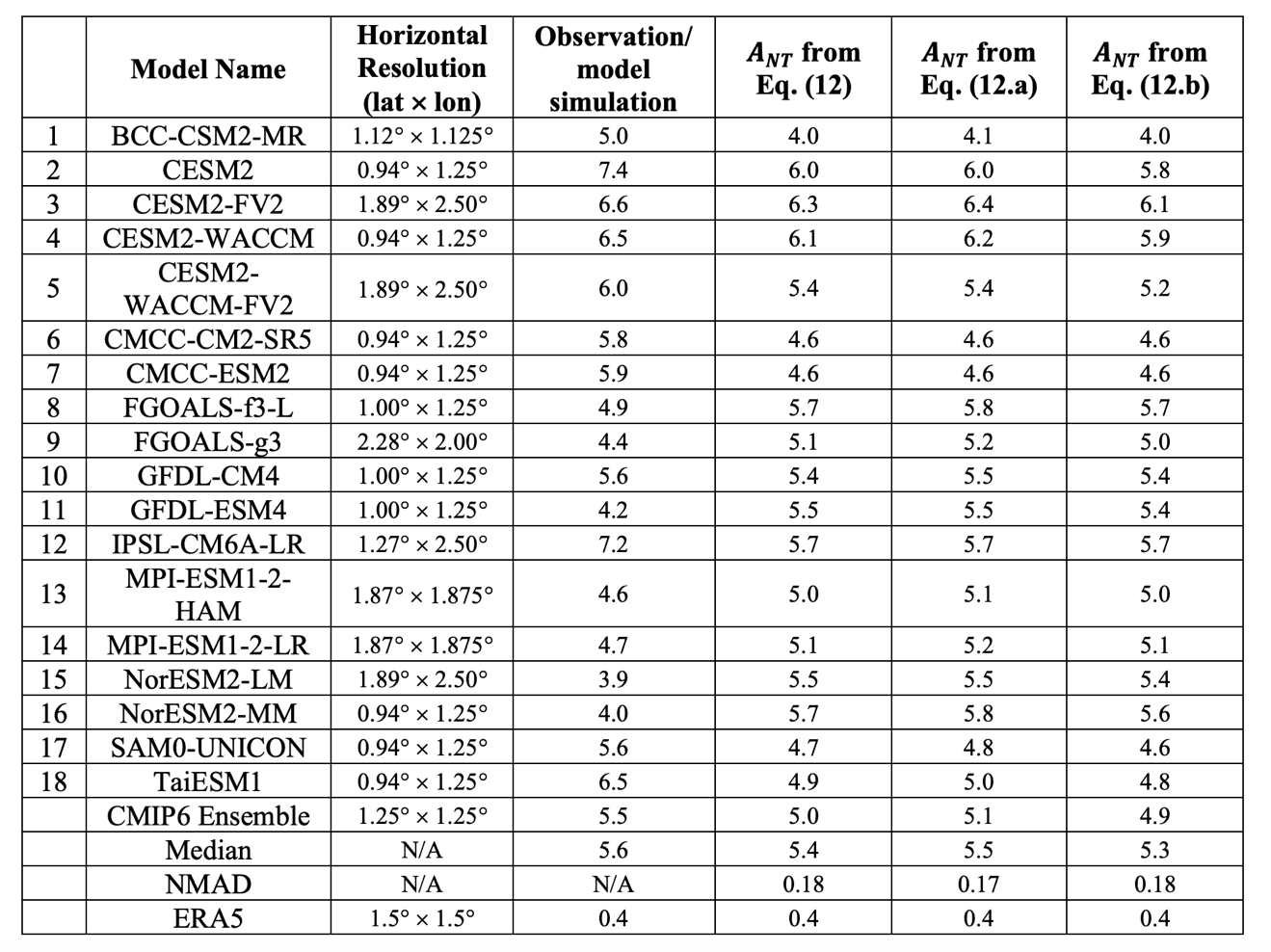

Supplement: nwae442_Supplemental_File [file nwae442_supplemental_file.docx]
